# A β‐ketoacyl carrier protein reductase confers heat tolerance via the regulation of fatty acid biosynthesis and stress signaling in rice

**DOI:** 10.1111/nph.17619

**Published:** 2021-07-30

**Authors:** Fei Chen, Guojun Dong, Fang Wang, Yingqi Shi, Jiayu Zhu, Yanli Zhang, Banpu Ruan, Yepin Wu, Xue Feng, Chenchen Zhao, Miing T. Yong, Paul Holford, Dali Zeng, Qian Qian, Limin Wu, Zhong‐Hua Chen, Yanchun Yu

**Affiliations:** ^1^ College of Life and Environmental Sciences Hangzhou Normal University Hangzhou 311121 China; ^2^ State Key Laboratory for Rice Biology China National Rice Research Institute Hangzhou 310006 China; ^3^ Institute of Insect Sciences Zhejiang University Hangzhou 310058 China; ^4^ College of Agronomy Qingdao Agricultural University Qingdao 266109 China; ^5^ School of Science Western Sydney University Penrith NSW 2751 Australia; ^6^ Hawkesbury Institute for the Environment Western Sydney University Penrith NSW 2751 Australia

**Keywords:** heat, hydrogen peroxide, lipids, membrane, *Oryza sativa*, signaling

## Abstract

- Heat stress is a major environmental threat affecting crop growth and productivity. However, the molecular mechanisms associated with plant responses to heat stress are poorly understood.
- Here, we identified a heat stress‐sensitive mutant, *hts1*, in rice. *HTS1* encodes a thylakoid membrane‐localized β‐ketoacyl carrier protein reductase (KAR) involved in *de novo* fatty acid biosynthesis. Phylogenetic and bioinformatic analysis showed that HTS1 probably originated from streptophyte algae and is evolutionarily conserved in land plants.
- Thermostable HTS1 is predominantly expressed in green tissues and strongly induced by heat stress, but is less responsive to salinity, cold and drought treatments. An amino acid substitution at A254T in HTS1 causes a significant decrease in KAR enzymatic activity and, consequently, impairs fatty acid synthesis and lipid metabolism in the *hts1* mutant, especially under heat stress. Compared to the wild‐type, the *hts1* mutant exhibited heat‐induced higher H_2_O_2_ accumulation, a larger Ca^2+^ influx to mesophyll cells, and more damage to membranes and chloroplasts. Also, disrupted heat stress signaling in the *hts1* mutant depresses the transcriptional activation of *HsfA2s* and the downstream target genes.
- We suggest that HTS1 is critical for underpinning membrane stability, chloroplast integrity and stress signaling for heat tolerance in rice.

## Introduction

Climate change is increasing the frequency of extreme heat events that affect crop growth and productivity (Lobell *et al*., 2011; Challinor *et al*., 2014). It has been estimated that average yield losses for major cereal crops caused by each degree Celsius increase in global mean temperatures could be up to 7.4% for maize, 6.0% for wheat, 3.2% for rice and 3.1% for soybean (Zhao *et al*., 2017). Extreme high temperature can induce heat stress in plants, which negatively affects metabolism, growth and development and may even lead to plant death (Wahid *et al*., 2007; Mittler *et al*., 2012). Therefore, it is essential to explore heat stress response (HSR) mechanisms and gain a better understanding of the molecular and genetic basis of plant HSRs, knowledge of which can facilitate the breeding of new crop varieties with increased tolerance to heat so as to sustain yield and quality under future climate conditions (Janni *et al*., 2020).

The major heat stress‐induced injuries in plants are a loss of membrane integrity and protein degradation, leading to an accumulation of reactive oxygen species (ROS) and programmed cell death (PCD) (Wahid *et al*., 2007; Bita & Gerats, 2013). The dominant pathways of plant HSRs have been well characterized with the identification of many heat stress‐responsive genes and their regulators in *Arabidopsis thaliana* and other plant species (Ohama *et al*., 2017; Li *et al*., 2018). Heat stress can rapidly activate transcriptional induction of heat shock transcription factors (HSFs) that upregulate the expression of heat‐responsive genes, including many heat shock proteins (HSPs) and genes encoding ROS‐scavenging enzymes (Ohama *et al*., 2017; Liu *et al*., 2021). This heat‐induced transcriptional response depends on multiple signal transduction pathways, such as Ca^2+^, ROS, nitric oxide (NO) and phospholipid signaling, which are all triggered by different heat sensors (Mittler *et al*., 2012; Zhu, 2016).

Lipids are major component of membranes that are critical for plant growth and development as well as responding to environmental challenges (Lim *et al*., 2017). Increasing evidence shows that plant responses to temperature changes are associated with the alteration of membrane lipid composition (Zhu *et al*., 2007; Higashi *et al*., 2015; Q. Li *et al*., 2015; Higashi & Saito, 2019; Liu *et al*., 2019). Several genes involved in lipid synthesis and remodeling play critical roles in plant heat tolerance. For example, *Arabidopsis* phospholipid:diacylglycerol acyltransferase1 (PDAT1), a diacylglycerol acyltransferase (DAG) that catalyzes the DAG‐to‐triacylglycerol (TAG) conversion, is involved in TAG production in plants under heat stress (Mueller *et al*., 2017). *Heat Inducible Lipase1* (*HIL1*) encodes a chloroplastic monogalactosyldiacylglycerol (MGDG) lipase that participates in plant response to heat stress via lipid remodeling (Higashi *et al*., 2018). In addition, plant fatty acid desaturase (FAD) enzymes are involved in heat stress responses due to their role in adjusting the desaturation status of membrane lipids, which is a critical determinant of membrane fluidity (Martiniere *et al*., 2011; Niu & Xiang, 2018; Ding *et al*., 2019). Moreover, several membrane lipids, such as phosphatidylinositol‐4,5‐bisphosphate (PIP2) and d‐*myo*‐inositol‐1,4,5‐trisphosphate (IP3), can directly serve as signaling molecules under heat stress (Mishkind *et al*., 2009; Zheng *et al*., 2012; Balogh *et al*., 2013).

Fatty acids are the basic components of membrane lipids. In plants, *de novo* fatty acids biosynthesis occurs in chloroplasts through the concerted actions of acetyl‐CoA carboxylase (ACCase) and fatty acid synthase (FAS). Biosynthesis is initiated by ACCase that carboxylates acetyl‐CoA to form malonyl‐CoA, which subsequently serves as the substrate for the elongation step through a series of condensation, dehydration and reduction reactions by FAS that result in the addition of carbons to form saturated fatty acids with 16 or 18 carbons (Ohlrogge & Jaworski, 1997). Then these 16 : 0 and 18 : 0 fatty acids can be desaturated and/or elongated to generate different fatty acids for multiple biological processes (Ohlrogge & Browse, 1995). Plants contain type II FAS consisting of β‐ketoacyl synthetase (KAS), 3‐ketoacyl‐acyl carrier protein (ACP) reductase (KAR), 3‐hydroxyacyl‐ACP dehydrase (HAD) and enoyl‐ACP reductase (ENR) (Ohlrogge & Jaworski, 1997). In *Arabidopsis*, a point mutation in *ENR* (*MOD1*) impaired fatty acid biosynthesis, causing pleiotropic effects on plant growth and development and premature cell death (Mou *et al*., 2000). An *Arabidopsis* mutant with a deficiency in β‐ketoacyl‐ACP synthase I (KSAI) exhibited stunted growth, reduced fertility and impaired chloroplast division (Wu & Xue, 2010). In rice, the *OskasI* mutant exhibits a short root phenotype and a large alteration in the composition and contents of fatty acids (Ding *et al*., 2015). *OsZl16* encodes an HAD in rice, and was found to be essential for chloroplast development via the regulation of fatty acid synthesis (Liu *et al*., 2018). Additionally, the gene *OsPLS4* is predicted to encode a KAR and is involved in the regulation of leaf senescence (Zhou *et al*., 2020). However, the role of FAS, especially KAR, in heat stress tolerance in rice is still unknown.

Rice is sensitive to high temperatures especially during its reproductive stage (Peng *et al*., 2004), and some genes involved in heat tolerance have been identified and characterized in this species. For example, Heat Tolerance At Seedling Stage (OsHTAS, a RING Finger ubiquitin E3 ligase) (Wei *et al*., 2013; Liu *et al*., 2016), Thermotolerant Growth Required1 (OsTOGR1, a nucleolar DEAD‐box RNA helicase) (Wang *et al*., 2016), an NAC transcription factor, OsNTL3 (Liu *et al*., 2020), and an RNA 5‐methylcytosine m^5^C methyltransferase, OsNSUN2 (Tang *et al*., 2020), all contribute to rice thermotolerance. *Thermo‐tolerance 1* (*OsTT1*), encoding an α2 subunit of the 26S proteasome, is reported as a dominant locus controlling heat tolerance in African rice (*Oryza glaberrima* Steud.) (X‐M. Li *et al*., 2015). In addition, transgenic rice overexpressing a receptor‐like kinase gene, *ERECTA* (*ER*), showed improved heat tolerance (Shen *et al*., 2015). Here, we report that *high temperature sensitive 1* (*HTS1*) encodes a functional KAR protein involved in *de novo* fatty acid biosynthesis that is essential for heat resistance in rice. Our results suggest that HTS1 is important for thermotolerance through the maintenance of membrane integrity and mediating heat‐signaling pathways in rice.

## Materials and Methods

### Plant materials and growth conditions

The *hts1* mutant was identified from a heat stress screen using an ethyl methanesulfonate (EMS)‐mutagenized rice population in the *Japonica* cultivar ‘Wuyunjing 7’. Rice plants were grown in a growth room under a 14 h : 10 h, light : dark cycle at 30°C : 24°C with 65% relative humidity (RH), or in an experimental field at the China National Rice Research Institute, Hangzhou (30°26′N, 120°19′E) under natural conditions.

Rice seeds were germinated and grown in IRRI nutrient solution (pH 5.8) in growth chambers at 28°C and 65% RH under a 14 h : 10 h, light : dark photoperiod. The nutrient solution was changed every 3 d. For heat tolerance testing of plants at the seedling stage, 2‐wk‐old seedlings grown at 28°C were transferred into a growth chamber at 45°C for 3 d, followed by 1 wk of recovery growth at 28°C, after which the survival of seedlings was evaluated; control plants remained at 28°C throughout the experiment. For heat tolerance testing of detached leaves, the first fully expanded leaves of 2‐wk‐old rice seedlings were detached and incubated at 45°C for 3 h in Petri dishes containing three‐layer filter paper wetted with 20 ml of deionized water, followed by 3 d of recovery at 28°C under continuous light (Yu *et al*., 2012). For the glutathione (GSH) and EGTA treatments, 20 ml of deionized water was replaced with solutions containing 400 μM GSH or 100 μM EGTA.

To evaluate the response of wild‐type and *hts1* to different stresses, the 2‐wk‐old wild‐type and *hts1* seedlings grown in solution culture were treated with cold stress (exposing plants to 4°C), salt stress (10 g l^−1^ NaCl) or drought stress (20% polyethylene glycol‐6000 (PEG)). For analysis of gene expression levels under different stresses, leaves from both stress‐treated and control wild‐type plants were sampled for a designated time.

### Map‐based cloning and complementation

Map‐based cloning of *HTS1* was performed with F_2_ populations generated from a cross between the *hts1* mutant and an *Indica* variety, 93‐11. A total of 1370 individual F_2_ seedlings exhibiting the *hts1* phenotype were screened for genetic mapping. *HTS1* was initially mapped to the long arm of Chromosome 4 between two simple sequence repeat (SSR) markers yp300 and yp398 using 20 individuals. For fine mapping, new sequence tagged site (STS) and SSR markers were generated based on genome polymorphisms between Nipponbare (subsp. *Japonica*) and 93‐11 (Yu *et al*., 2008). Candidate genes were predicted using the Rice Genome Annotation Project databases (http://rice.plantbiology.msu.edu/). Genomic DNA fragments of candidate genes were amplified from wild‐type and mutant plants, which were then sequenced and compared to identify the *hts1* mutation.

For complementation of the *hts1* mutant, a 7683 bp genomic DNA fragment containing the entire *HTS1* coding sequence, a 1583 bp upstream region and an 879 bp downstream sequence were amplified using Q5 DNA polymerase (NEB) from the wild‐type genomic DNA and cloned into the binary vector, pCAMBIA1301. The binary construct was then introduced into *Agrobacterium tumefaciens* strain GV3101 and transformed into *hts1* mutant callus as described by Hiei *et al*. (1994). The primers used in this study are listed in Supporting Information Table S1.

### Bioinformatic analysis

The orthologs of HTS1 in green plants were identified using the 1KP database (One Thousand Plant Transcriptomes, 2019) and the NCBI database by Blastp analysis (http://blast.ncbi.nlm.nih.gov/Blast.cgi). Phylogenetic analysis was performed using FastTree (http://www.microbesonline.org/fasttree/) through a maximum‐likelihood method as described by Zhao *et al*. (2019). Alignment of protein sequences selected from the representative species were aligned using Jalview software with Mafft (https://mafft.cbrc.jp/alignment/software/) and Logo analysis (http://www.weblogo.berkeley.edu/logo.cgi) according to Feng *et al*. (2020). Protein domain analysis was performed by CCD‐Search based on the NCBI conserved domain database (https://www.ncbi.nlm.nih.gov/cdd). The putative chloroplast transit peptide of HTS1 was predicted using ChloroP (http://www.cbs.dtu.dk/services/ChloroP/) (Emanuelsson *et al*., 1999).

### Quantitative real‐time PCR

Total RNA from different tissues was extracted using the RNeasy Plant Mini Kit (Qiagen), and first‐strand cDNA synthesis was performed using ReverTra Ace qPCR RT Master Mix with gDNA remover (Toyobo, Osaka, Japan). qRT‐PCR analysis was performed with TB Green Premix Ex Taq (Takara, Kusatsu, Japan) with a CFX96 Real‐Time PCR Detection System (Bio‐Rad). The transcript data were normalized using the rice actin gene *LOC_Os03g50885* as an internal control.

### Histochemical GUS staining

To construct pHTS1::GUS, a 1583 bp genomic DNA fragment immediately before the start codon of *HTS1* was amplified by PCR using the primer pair pGUS‐F and pGUS‐R (Table S1) and cloned into the pCAMBIA1301 vector using the *Kpn*I and *Nco*I restriction sites. The resulting construct was then introduced into wild‐type plants by *Agrobacterium tumefaciens*‐mediated transformation. T2 transgenic positive plants were used for analysis of GUS activity by the method described by Xiong *et al*. (2010).

### Subcellular localization of HTS1 and its close homologs in rice

For subcellular localization of HTS1 and its close homologs in rice, the coding region of *HTS1* and its close homologs without the stop codon was amplified from the wild‐type cDNA library by PCR using specific primers (Table S1). Then, the genes were cloned into a modified pCAMBIA1300 vector containing a cauliflower mosaic virus (CaMV) 35S::GFP cassette (He *et al*., 2018). The fusion constructs and the empty vector were transferred into rice protoplasts according to Chen *et al*. (2018) and green fluorescent protein (GFP) fluorescence was observed using confocal laser‐scanning microscopy (CLSM) (LSM710; Zeiss) after overnight incubation in the dark.

### Chloroplast subfraction isolation and western blot analysis

Intact chloroplasts and thylakoid, stroma and envelop subfractions were isolated as described in our previous study (Chen *et al*., 2018). Western blotting was performed according to standard techniques using specific antibodies. Immunodetection of proteins on a polyvinylidene fluoride (PVDF) membrane was performed using an enhanced chemiluminescence kit (Invitrogen), and analyzed by a ChemiDoc Touch imaging system (Bio‐Rad).

### Heat stability of HTS1 protein

The heat stability of the HTS1 protein was assessed as previously described (Rao *et al*., 2004). Aliquots (300 μl) of purified protein (50 μg l^−1^) were incubated at 25°C (control) or 45°C (heat treatment) for 15, 30 and 45 min. After incubation, samples were centrifuged at 15 000 ***g*** for 15 min and equal volumes of supernatants were loaded for western blot analysis. The heat‐instable protein rubisco activase is used as a negative control (Ristic *et al*., 2007).

### Cell physiology

Chlorophyll contents in leaves from 2‐wk‐old wild‐type and *hts1* mutant plants were measured as previously described (Chen *et al*., 2018). Cell death was observed using trypan blue staining as previously described (Wu *et al*., 2015). H_2_O_2_ accumulation was determined by staining with 3,3′‐diaminobenzidine (DAB) (Chen *et al*., 2010), or quantified using an Amplex Red hydrogen peroxide/peroxidase assay kit (Invitrogen) following the manufacturer’s instructions. The antioxidant enzyme activities and malondialdehyde (MDA) contents were determined according to Chen *et al*. (2010).

Ion leakage was measured as previously described (Shen *et al*., 2015). Briefly, three leaves of 2‐wk‐old rice seedlings harvested after 0, 12, 24 and 48 h of heat stress were trimmed into small pieces and placed in glass tubes containing 10 ml deionized water and incubated overnight at 25°C with shaking (*c.* 100 rpm). Initial ion leakage (*I*_0_) was determined with a conductivity meter (Mettler Toledo, OH, USA). Total ion leakage (*I*_t_) was measured after 15 min of boiling. Relative ion leakage was expressed as a percentage (*I*_0_/*I*_t_).

### Transmission electron microscopy and confocal microscopy

Transmission electron microscopy (TEM) and observations of leaf autofluorescence were performed according to our previous study (Chen *et al*., 2018). Leaf samples from 2‐wk‐old rice seedlings with or without heat treatment were collected for TEM (JEM‐1230 EX; JEOL, Japan), or for leaf fluorescence imaging by a low‐light cooled CCD imaging apparatus (CA2048B; Roper Scientific, Trenton, NJ, USA). Cellular ROS production was determined by confocal imaging using the fluorescent dye 2′,7′‐dichlorodihydrofluorescein diacetate (H2DCFDA; Invitrogen) as described previously (Cai *et al*., 2021). Mesophyll cells collected from the first expanded leaves of 2‐wk‐old rice plants were used for CLSM (Leica, Wetzlar, Germany).

### Enzyme activity

To obtain purified HTS1‐GST protein, the coding sequence of *HTS1* was amplified from wild‐type or *hts1* mutant plants using the primers HTS1‐GST‐F and HTS1‐GST‐R (Table S1) and cloned into the vector pET30a. The two resulting constructs and the empty vector were then introduced into *Escherichia coli* strain BL21. Protein induction and purification was performed according to Chen *et al*. (2018). KAR activity was determined based on monitoring the changes in NADPH absorption at 340 nm as described previously (Veyron‐Churlet *et al*., 2010).

### Fatty acid analysis

Leaf samples from 2‐wk‐old rice plants with or without heat treatment were collected for analysis of lipids and fatty acids. Lipid profiling was performed at Qingdao Kechuang Quality Analysis Ltd (Shandong, China). Total lipids were extracted with methanol : dichloromethane (2 : 1, v/v) and the extracts were analyzed using a UHPLC Ultimate 3000 system coupled with a Q‐Exactive HF Mass Spectrometer (Thermo Fisher Scientific, San Jose, CA, USA). Fatty acid methyl esters were prepared according to Wu & Xue (2010). Methyl heptadecanoate (AccuStandard Inc., USA) stock solution was added to obtain a final concentration of 25 ppm as an internal reference. GC‐MS was performed using an Agilent 5975 inert GC/MS system (Agilent, CA, USA).

### Ion flux measurements

Net Ca^2+^ fluxes of cells were measured using ion‐selective vibrating microelectrodes (MIFEs) as described by Chen *et al*. (2005). Ca^2+^ flux measurements were conducted with mesophyll cells from leaves of 2‐wk‐old rice plants placed in the measuring buffer for 10 min as the control, and after a further 20 min at 45°C as the heat treatment.

### Statistical analysis

Statistical analyses were performed with the Data Processing System (DPS) statistical software package (Tang & Zhang, 2013) using ANOVA followed by Student’s *t*‐test and Duncan’s multiple range test to evaluate significant effects of the treatments at a significance level of *P* < 0.05.

## Results

### The *hts1* mutant is hypersensitive to heat stress

**Fig. 1:**
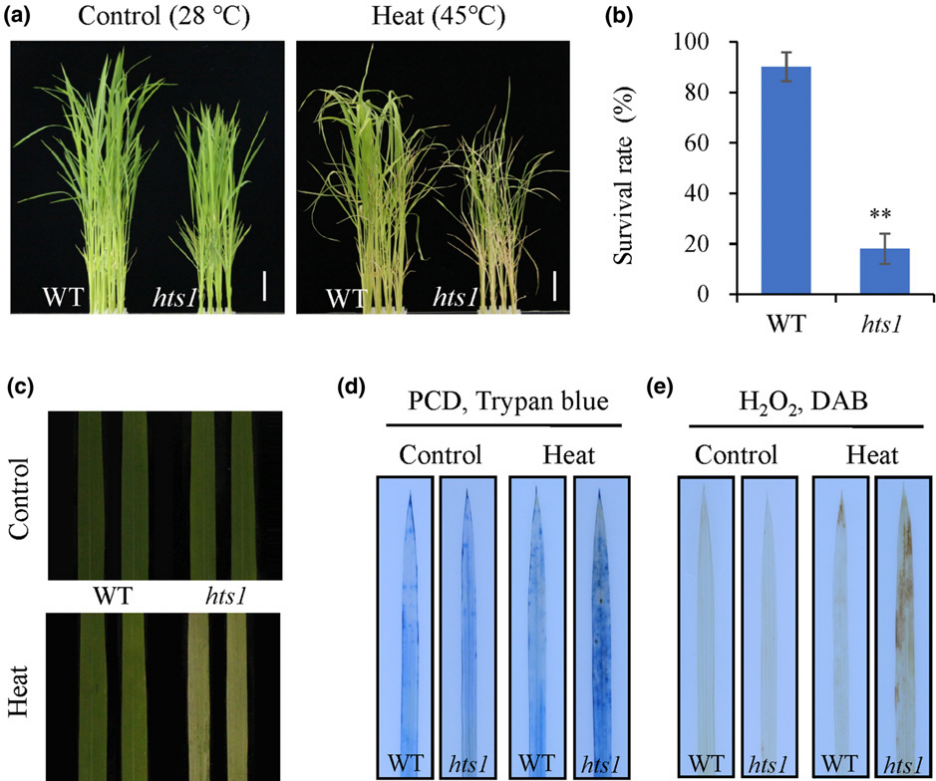
*hts1* is a heat‐sensitive mutant. (a) Phenotypes of 2‐wk‐old rice seedlings before and after heat treatments. Two‐week‐old wild‐type (WT) and *hts1* mutant seedlings grown at 28°C were transferred to 45°C for 3 d and then photographed after recovering at 28°C for 2 d. Bars, 20 mm. (b) Survival rates of WT and *hts1* mutant seedlings after recovery from heat treatment. Data are means ± SD (*n* = 3). **, *P* < 0.01. (c) Heat‐sensitive phenotype in detached leaves of the *hts1* mutant in comparison with the WT. (d, e) Visualization of programmed cell death (PCD) (d) and H_2_O_2_ accumulation (e) using DAB and trypan blue staining of leaves from 2‐wk‐old seedlings grown at 28°C and treated with heat (45°C) for 0 and 48 h.

After screening an EMS‐mutagenized library of rice, we isolated a number of mutant plants with altered sensitivity to heat stress. The mutant, *high temperature sensitive 1* (*hts1*), exhibits a severe heat sensitive phenotype at the seedling stage. The mutant plants showed earlier leaf curling and wilting than the wild‐type when subjected to a 45°C heat treatment. Approximately 90% of wild‐type plants survived 72 h of heat treatment, whereas the survival rate of the mutant plants was only 18.1% (Fig. 1a,b); the mutants were also less green than the controls (Fig. 1c). Under normal growth conditions, the mutant plants also showed some mild morphological defects, including slightly reduced Chl contents, plant heights, tiller numbers, seed setting rates and grain sizes (Figs S1, S2). To further explore whether *HTS1* is specifically involved in heat tolerance, we examined the effects of *HTS1* on different abiotic stresses. No obvious difference between wild‐type and *hts1* mutant seedlings was observed due to salt, cold or drought treatments (Fig. S3a).

We then investigated whether *HTS1* affects heat stress‐induced ROS production and cell damage. Staining with trypan blue was more intense in detached leaves from *hts1* plants compared to the wild‐type after the plants had been exposed to a heat treatment, indicating the presence of more heat‐induced cell death in the mutant (Fig. 1d). Malondialdehyde contents, resulting from lipid peroxidation of polyunsaturated fatty acids, and H_2_O_2_ contents were significantly increased in *hts1* leaves after exposure to the heat treatment (Figs 1e, S4a,b). However, the heat‐induced H_2_O_2_ accumulation and the sensitivity of the mutant were substantially reduced by exogenous application of 400 µM reduced GSH (an H_2_O_2_ scavenger) (Fig. S5). We also found that the heat treatment significantly increased the activities of the antioxidant enzymes, APX and CAT, in wild‐type leaves, but not in those from the *hts1* mutant (Fig. S4c). Together, these results clearly demonstrated that *HTS1* contributes to heat stress resistance and oxidative stress response in rice.

### *HTS1* encodes a β‐ketoacyl carrier protein reductase conserved in land plants

**Fig. 2:**
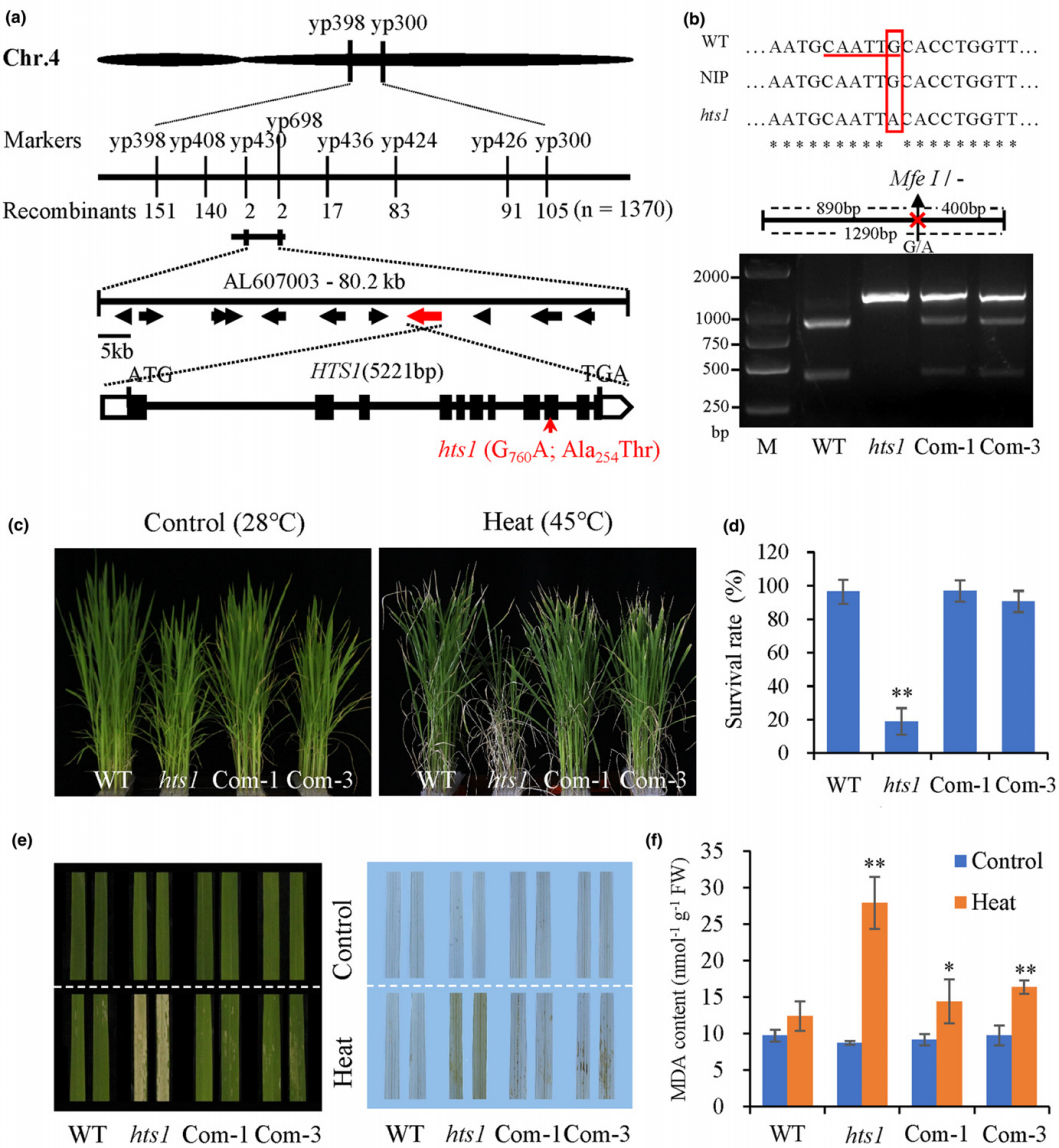
Cloning of the *HTS1* gene and verification of *HTS1* function. (a) Map‐based cloning of the *HTS1* gene and a schematic representation of the *HTS1* gene structure (bottom). The *HTS1* locus was mapped primarily to the long arm of rice (*Oryza sativa*) chromosome 4 (Chr. 4) between markers yp398 and yp300. It was subsequently narrowed to an 80.2 kb region between yp430 and yp698 within the bacterial artificial chromosome clone AL607003 using 1370 homozygous mutant plants. The predicted ORFs in this region are highlighted with arrows. The *HTS1* gene structure, including exons (black boxes), introns (black lines), the UTR (open boxes), ATG start and TGA stop codons, and a single base substitution (G to A) in *hts1* are indicated. (b) The presence of normal transcripts of *HTS1* in transgenic line *hts1*‐Com (*hts1* with complemented expression of *HTS1*) was confirmed by PCR followed by restriction enzyme digestion. A 1290‐bp DNA fragment around the mutation is amplified with the specific primers (yp2243‐F and R) and then digested with the restriction enzyme *Mfe*I. An *Mfe*I restriction site (underlined) was abolished by the G‐to‐A substitution (red box) in the mutant sequence. (c) Iamges of 2‐wk‐old plants of the wild‐type, *hts1* and *hts1*‐Com lines (Com‐1 and Com‐2) grown at 28°C and after 72 h of treatment at 45°C and 2 d recovery at 28°C. (d) Survival rates of the wild‐type, *hts1* and the *hts1*‐Com lines after exposure to heat treatment (45°C, 72 h). (e) Images of the heat‐challenged detached leaves (left panel) from four different genotypes described in (c). DAB staining of these detached leaves is shown in the right panel. (f) MDA contents of the wild‐type, *hts1* and hts1‐Com plants before and after exposure to heat treatment (45°C, 48 h). Data are means ± SD (*n* = 3). *, *P* < 0.05; **, *P* < 0.01.

To identify the *HTS1* locus, an F_2_ population was generated from a cross between *hts1* and the *Indica* variety, 93‐11. Segregation analysis revealed that the mutant phenotype is controlled by a single recessive gene. Using 1370 F_2_ recessive plants and PCR‐based molecular markers, we mapped the *HTS1* locus to an 80.2 kb interval between two STS markers, yp430 and yp698, on the long arm of Chromosome 4 (Fig. 2a). This region includes eleven annotated candidate open reading frames (ORFs) (http://rice.plantbiology.msu.edu/). We then amplified and sequenced these genes from wild‐type and *hts1*; only a single nucleotide substitution (G‐to‐A) in Exon 9 of *LOC_Os04g30760* was identified that converted alanine (Ala) to threonine (Thr) (Fig. 2a). To confirm that the mutation in *LOC_Os04g30760* is responsible for the *hts1* phenotype, a genetic complementation test was performed by introducing a 7.6 kb genomic fragment of the candidate gene containing its entire coding region, together with 1583 bp upstream and 879 bp downstream sequences into the *hts1* plants. The presence of *HTS1* in transgenic lines was verified by PCR followed by restriction enzyme digestion of an *Mfe*I restriction site, which was abolished by the single nucleotide substitution in *hts1* (Fig. 2b). The resulting transgenic‐positive (designated as *hts1*‐Com) lines were restored to the wild‐type phenotype under heat stress conditions (Fig. 2c–e). Growth at the seedling and reproductive stages was also restored in the *hts1*‐Com lines (Fig. S6). These results showed that the heat hypersensitivity of the *hts1* mutant is caused by the mutation in the *LOC_Os04g30760* gene.

**Fig. 3:**
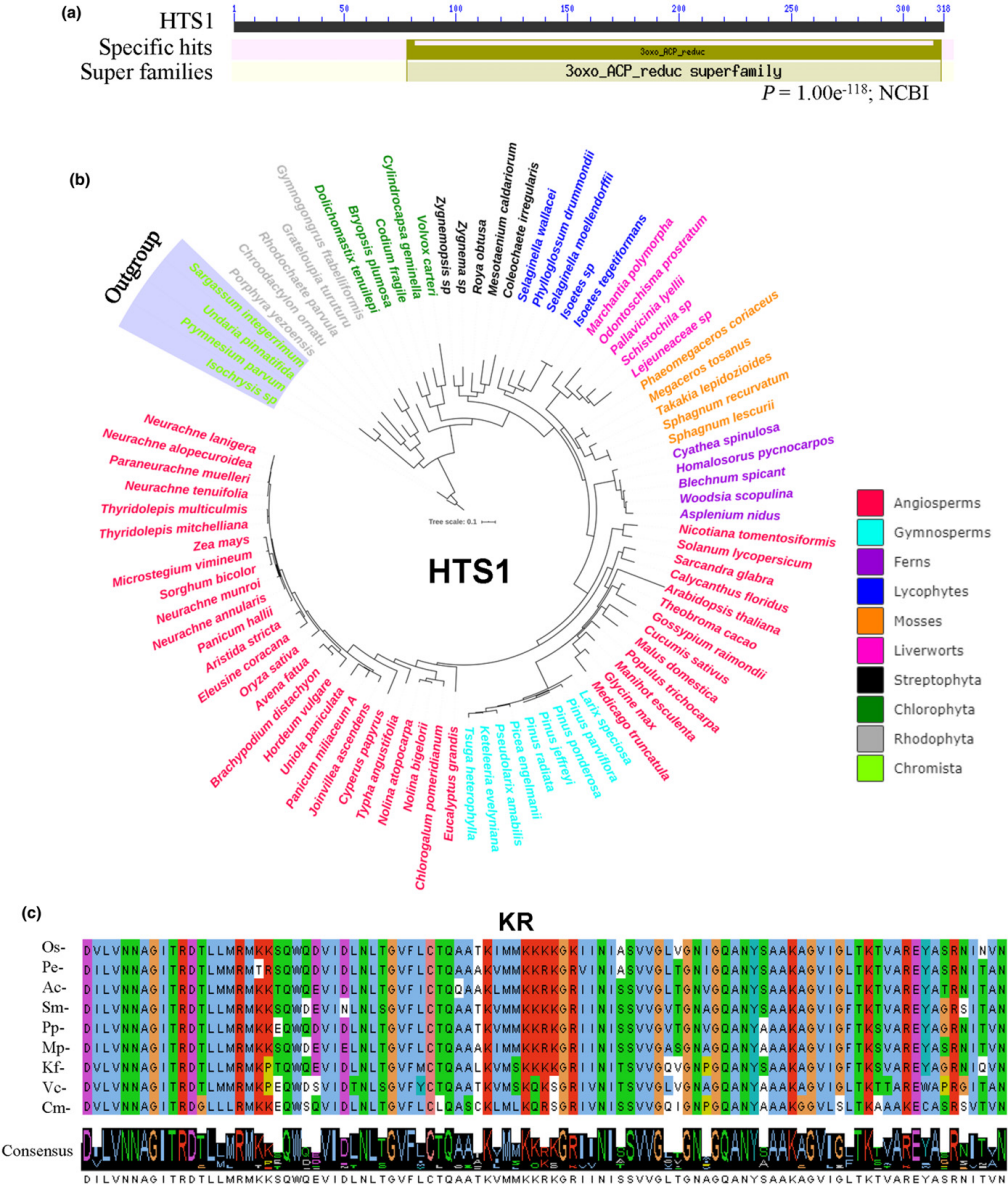
Phylogenetic trees and conserved domain of HTS1 proteins in plants and algae. (a) Conserved domain of HTS1 was predicted in the NCBI database (https://www.ncbi.nlm.nih.gov/cdd). (b) Phylogenetic trees of HTS1 proteins in representative species of major lineage of plants and algae. The maximum‐likelihood method was used to construct the trees. Clades are indicated by different colors. (c) Conserved domain alignment of HTS1s. KR, ketoreductase. Os, *Oryza sativa*; Pe, *Picea engelmanii*; Ac, *Azolla caroliniana*; Sm, *Selaginella moellendorffii*; Pp, *Physcomitrella patens*; Mp, *Marchantia polymorpha*; Kf, *Klebsormidium flaccidum*; Vc, *Volvox carteri*; Cm, *Cyanidioschyzon merolae*.

The full‐length ORF of *HTS1* (957 bp) encodes a 318 amino acid (aa) protein with a calculated molecular mass of 32.6 kDa. HTS1 contains a putative chloroplast transit peptide at the N‐terminus and a conserved domain of the KAR superfamily at amino acid positions 78–317 (Fig. 3a). Recently, this protein was found to be chloroplast‐located (annotated as PLS4) and to play a role in leaf senescence in rice (Zhou *et al*., 2020). Here we refer to PLS4 as ‘HTS1’. The rice genome contains two homologs of HTS1, and phylogenetic analysis revealed that HTS1 is closely related to GRMZM2G099696 in maize and Sb6g010860 in sorghum (Fig. S7). Moreover, analysis using the 1KP database showed that 76% of the 1322 plant and algal species have predicted protein orthologs of HTS1 (Figs 3b, S8; Table S2). Amino acid alignment and Logo analysis of the protein domains revealed conserved sequence similarity of HTS1s among representative green plant species. However, there were significant sequence differences in the sequences of the HTS1s in rhodophyte and chlorophyte algae as compared with those in streptophyte algae and land plants (Fig. 3c), indicating that HTS1 has evolved from streptophyte algae and is evolutionarily conserved in land plants.

### HTS1 is localized to the thylakoid membrane and induced by heat treatment

**Fig. 4:**
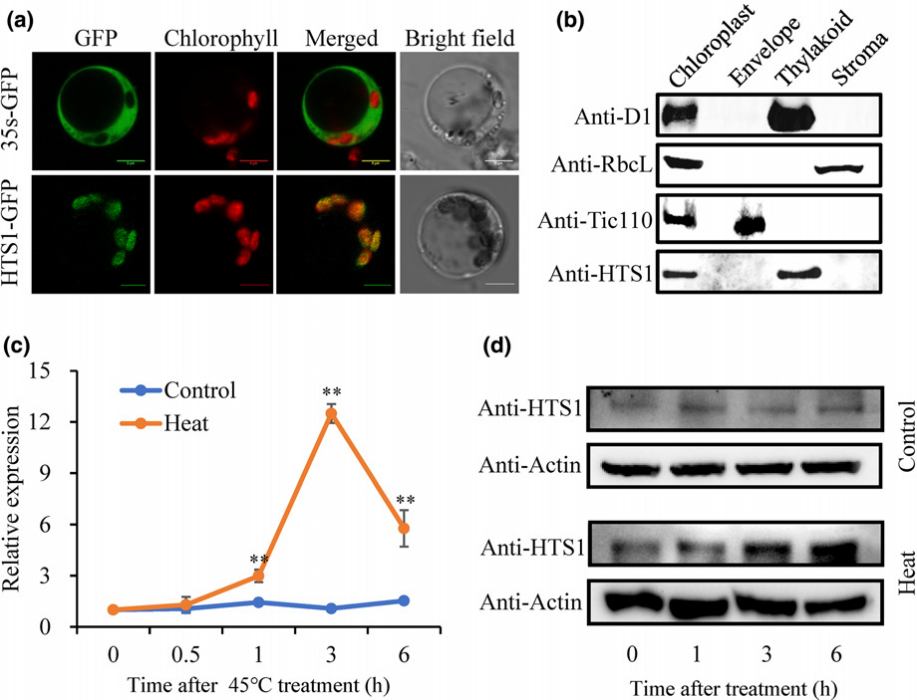
HTS1 is a thylakoid membrane‐localized protein and is in response to heat stress in rice (*Oryza sativa*). (a) Confocal imaging showing chloroplast targeting of HTS1. 35S::GFP (upper panel) and 35S::HTS1‐GFP (lower panel) transiently expressed in rice protoplast cells. Left to right, GFP fluorescence, chloroplast autofluorescence, merged images and bright field images. Bars, 5 μm. (b) Suborganelle localization of the HTS1 protein in the chloroplast. Intact chloroplasts were isolated from WT leaves then separated into thylakoid membrane, envelope and stroma fractions. Polyclonal antibodies against D1 (thylakoid marker), RbcL (stroma marker) and Tic110 (envelope marker) were used as markers. (c, d) The mRNA (c) and protein (d) levels of HTS1 in wild‐type leaves after 45°C heat treatment. The protein levels of HTS1 were measured by western blotting. Equal protein loading was confirmed with antiserum against actin.

To determine the subcellular localization of HTS1, a 35S:HTS1‐GFP construct was introduced into rice mesophyll protoplasts. Confocal laser‐scanning microscopy revealed that the GFP signal of the HTS1‐GFP fusion protein colocalizes with Chl fluorescence (red) in the protoplasts (Fig. 4a). To further determine the subplastidic localization of HTS1, we raised anti‐HTS1‐specific polyclonal antibodies using recombinant full‐length HTS1 protein as the antigen. HTS1 antibodies specifically recognized both endogenous HTS1 as a 32.6 kDa band in the total protein extracts from the wild‐type and *hts1* mutant and recombinant GST‐HTS1 fusion protein as a clear 58.6 kDa protein band (Fig. S9). Immunoblot analysis of total protein extracts from different fractions of the wild‐type chloroplasts verified that the subplastidic localization of HTS1 is similar to that of D1 protein (Fig. 4b), a known thylakoid membrane protein encoded by the *psbA* gene in chloroplast genomes (Zhang *et al*., 1999).

Quantitative RT‐PCR (qRT‐PCR) showed that *HTS1* is mainly expressed in green tissues, with highest expression levels in leaves and leaf sheaths, followed by panicles (Fig. S10a). This expression pattern was confirmed by strong GUS staining in leaves, leaf sheaths and young glumes (Fig. S10b). Moreover, we also examined the expression of *HTS1* under abiotic stresses. qRT‐PCR showed that heat treatment significantly increases the transcript level of *HTS1*, reaching peak expression (12.5‐fold) after 3 h at 45°C (Fig. 4c). However, transcript levels of *HTS1* were less induced by cold and drought (PEG treatment) stress (up to 2.5‐fold at 0.5 h) and by salt stress (up to 5‐fold at 8 h) (Fig. S3b). Western blot analysis further revealed that heat treatment gradually increases HTS1 protein levels with a peak at 6 h after the start of the treatment, indicating that HTS1 is a heat‐inducible protein (Fig. 4d).

### HTS1 has KAR activity and exhibits high thermal stability

**Fig. 5:**
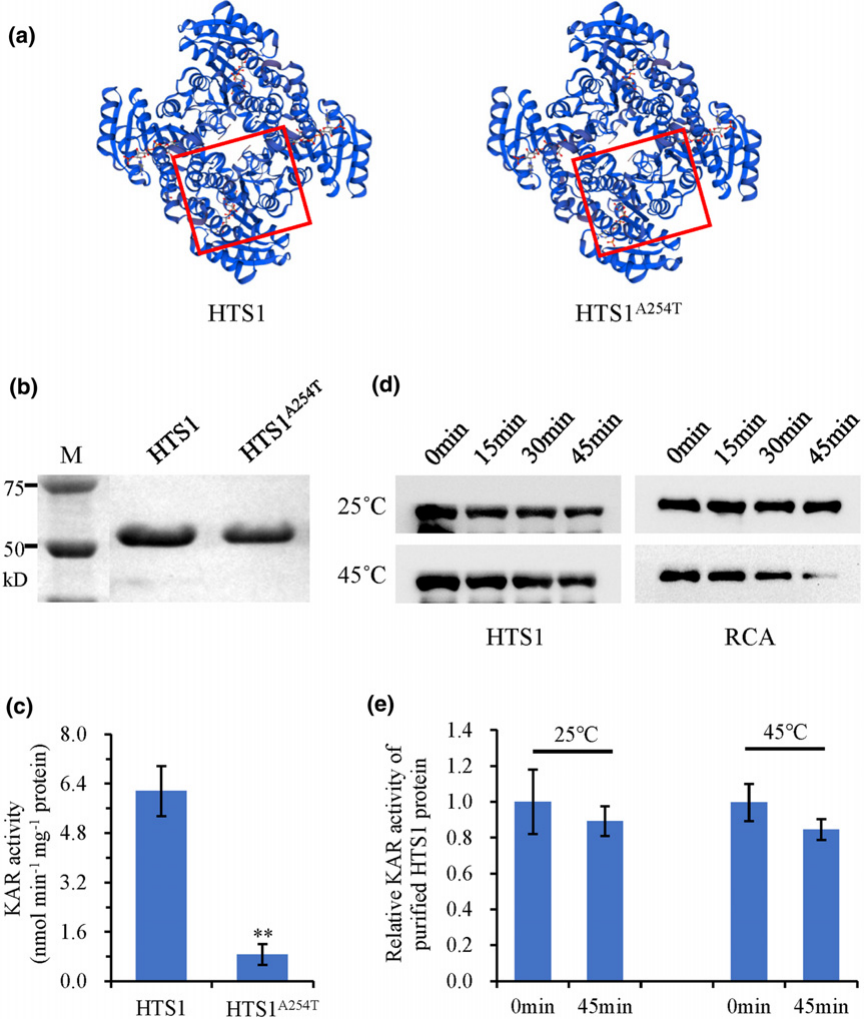
The predicted protein structures and enzymatic activity of 3‐oxoacyl‐acyl‐carrier‐protein reductase (HTS1) and HTS1^A254T^. (a) Three‐dimensional models of HTS1 and HTS1^A254T^ predicted by Swiss‐Model. (b) The purification of GST‐tagged HTS1 and mutant proteins from *Escherichia coli*. (c) Enzyme activity assay of HTS1 *in vitro*. (d) Western blot of purified HTS1 protein incubated at 25°C (control) and 45°C for the indicated times. Purified Rubisco activase (RCA) protein was used as the control (Ristic *et al*., 2007). (e) Enzyme activity of purified HTS1 protein incubated at 25°C (control) and 45°C. Data represent means ± SD (*n* = 3). **, *P* < 0.01.

KAR is a key enzyme in the type II fatty acid elongation cycle that reduces 3‐ketoacyl‐ACP to 3‐hydroxyl (Brown *et al*., 2009). Prediction of protein structure showed that the mutation of the Thr254 amino acid residue in the conserved domain of KAR may cause conformational alteration of the HTS1 protein (Fig. 5a). Therefore, we expressed the N‐terminal fusion of HTS1 and mutant (hereafter referred to A254T) with glutathione *S*‐transferase (GST) using an *E*. *coli* expression system (Fig. 5b) and KAR activity was examined. High catalytic activity was detected in GST‐HTS1 after introducing acetoacetyl‐CoA and NADH, whereas the activity of GST‐HTS1[A254T] was significantly reduced by 85.7% (Fig. 5c).

We further examined the stability of HTS1 under heat stress by assessing its ability to remain soluble and maintain enzyme activity after treatment at 45°C. Western blot analysis showed that both the recombinant and the endogenous HTS1 protein are stable in the soluble fraction after the heat treatments. By contrast, the control rubisco activase, a heat‐unstable protein (Ristic *et al*., 2007), showed a sharp decline in the soluble fraction after 30 min of heat treatment (Figs 5d, S11) indicating that HTS1 is relatively stable at 45°C. Moreover, the purified HTS1 protein retained 84.5% enzyme activity after heat treatment as compared to the control, corroborating the results of the western blot experiments (Fig. 5e).

### The *hts1* mutant was defective in fatty acid biosynthesis

**Fig. 6:**
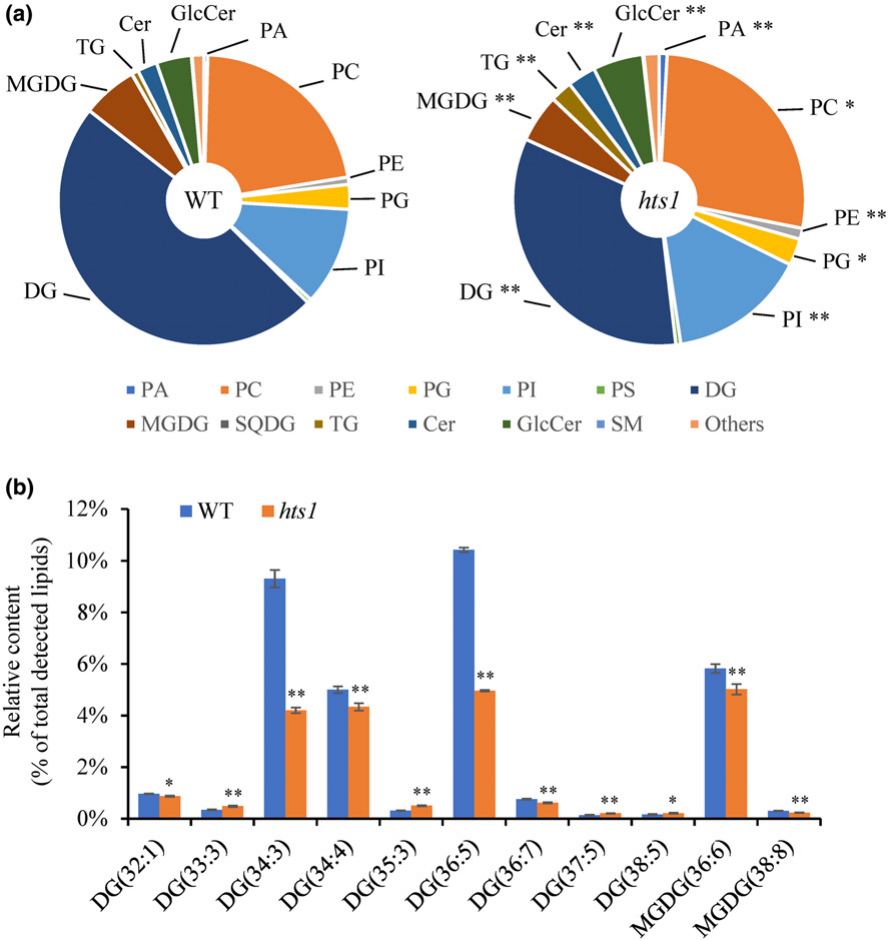
Lipidomic analysis of leaves of wild‐type and *hts1* mutant rice (*Oryza sativa*). (a) Lipid compositions in leaves from 2‐wk‐old wild‐type and *hts1* seedlings. Lipid classes are presented as the percentage of the total amount of all detected lipid classes. (b) Relative abundance of different molecular species of glycerolipids (DG and MGDG) in the wild‐type and *hts1* mutant. Data represent means ± SD (*n* = 3). **, *P* < 0.01; *, *P* < 0.05. Cer, ceramides; DG, diglyceride; GlcCer, glucosylceramide; MGDG, monogalactosyldiacylglycerol; PA, phosphatidic acid; PC, phosphatidylcholine; PE, phosphatidylethanolamine; PG, phosphatidylglycerol; PI, phosphatidylinositol; PS, phosphatidylserine; SM, sphingomyelin; SQDG, sulfoquinovosyldiacylglycerol; TG, triglyceride.

We then examined whether fatty acid biosynthesis and lipid metabolism are impaired in the *hts1* mutant due to reduced KAR activity. Lipidomic analysis showed that *hts1* has significantly lower glycerolipids (diglyceride (DG) and MGDG) than the wild‐type plants. By contrast, the levels of glycerophospholipids, such as phosphatidic acid (PA), phosphatidylcholine (PC), phosphatidylglycerol (PG), phosphatidylethanolamine (PE) and phosphatidylinositol (PI), showed slight increases in the mutant (Fig. 6a; Table S3). Interestingly, the compositions of these glycerolipids showed that there was a highly significant decrease in the 34 : 3 and 36 : 5 components of the DGs. The decreased levels of MGDG were mainly explained by a small reduction in 36 : 6 and 38 : 8 species (Fig. 6b; Table S4). Further analysis of the fatty acids showed that the contents of the two most abundant fatty acids, C16 : 0 (palmitic acid) and C18 : 3 (linolenic acid), were significantly reduced in the *hts1* mutant compared to the wild‐type. By contrast, the levels of fatty acids in HTS1‐complemented plants were similar to those observed in wild‐type plants (Table 1). Heat treatment significantly reduced the fatty acid content in leaves, but the wild‐type still had 46.9% more fatty acids than *hts1* after 24 h. In particular, the heat‐induced total fatty acid reduction was mainly due to the decrease of C16 : 0, C18 : 0 and C18:3 species (Table 1).

**Table 1:** Changes in fatty acid compositions of wild‐type (WT), *hts1* and *hts1*‐Com (Com‐1) plants grown at 28°C or followed by 24 h of heat treatment at 45°C.

Fatty acid	28°C			45°C		
	WT	*hts1*	Com‐1	WT	*hts1*	Com‐1
C16 : 0	18.2 ± 0.27	13.4 ± 0.36*	17.6 ± 0.51	11.8 ± 0.37	6.78 ± 0.82*	12.57 ± 0.24
C16 : 1	1.36 ± 0.04	1.20 ± 0.10	1.53 ± 0.19	1.21 ± 0.04	0.94 ± 0.04*	1.09 ± 0.03
C18 : 0	7.54 ± 0.07	5.29 ± 0.94*	6.39 ± 0.82	4.42 ± 0.45	2.38 ± 0.83*	4.48 ± 0.45
C18 : 2	5.14 ± 0.44	4.91 ± 0.41	5.30 ± 0.32	3.51 ± 0.39	3.42 ± 0.15	3.79 ± 0.47
C18 : 3	27.1 ± 1.44	21.5 ± 1.33*	27.9 ± 0.78	24.2 ± 1.04	18.5 ± 1.09*	23.58 ± 1.13
Total	58.6 ± 1.13	46.3 ± 0.92	57.11 ± 0.93	45.1 ± 2.30	30.7 ± 0.66	43.2 ± 2.24

Data are means ± SD (μg mg^−1^ DW; *n* = 3).

* Significant differences at *P* < 0.05 compared to the WT.

### *HTS1* deficiency accelerates loss of membrane integrity under heat stress

**Fig. 7:**
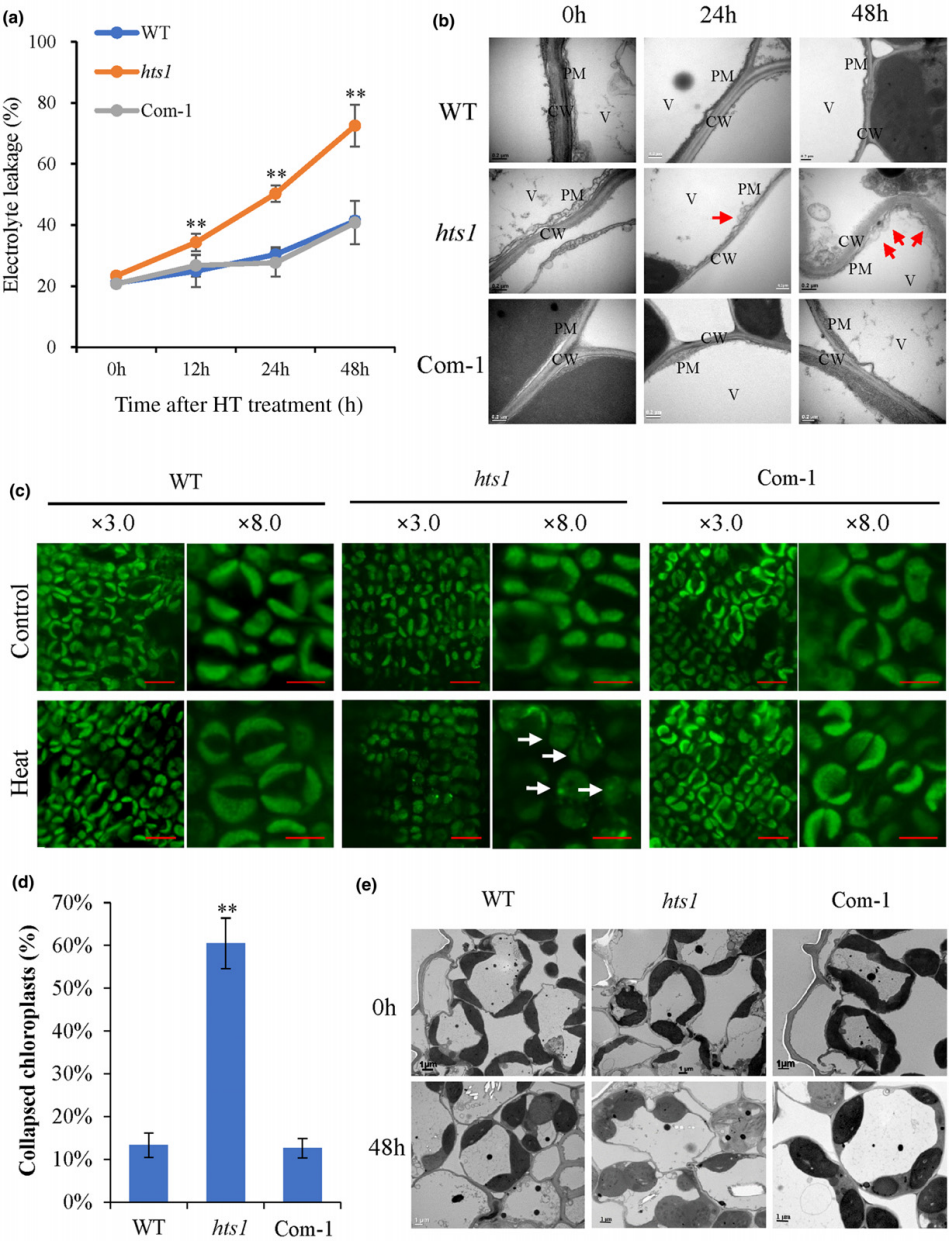
Disturbance of *HTS1* expression accelerates plasma membrane disruption and chloroplast degradation under heat treatment. (a) Change of cellular ion leakage of the fully expanded leaves from WT, *hts1* and *hts1*‐Com1 plants challenged with heat treatment (45°C) for the indicated time. Data are means ± SD (*n* = 3). **, *P* < 0.01. (b) TEM subcellular observation of the plasma membrane of the 2‐wk‐old WT, *hts1* and *hts1*‐Com1 plants treated at 45°C for 0, 24 and 48 h. Red arrows indicate the plasma membrane blebbing in the *hts1* mutant. PM, plasma membrane; CW, cell wall; V, vacuole. Bar, 0.2 μm. (c) Confocal images of mesophyll cells of the 2‐wk‐old WT, *hts1* and *hts1*‐Com1 plants treated at 45°C for 0 and 2 h. White arrows indicate disrupted chloroplasts. Bars, 10 μm (×3.0) and 5 μm (×8.0). (d) Quantification of the occurrence of chloroplast disruption (swelling and collapsing) in mesophyll cells after 2 h of heat treatment. Data are means ± SD (*n* = 5 with 30–50 cells). **, *P* < 0.01. (e) TEM observation of chloroplast collapse of the 2‐wk‐old WT, *hts1* and *hts1*‐Com1 plants treated at 45°C for 0 and 48 h. Bars, 1 μm.

Considering the important role of fatty acids in cellular membrane formation, we investigated the effect of *HTS1* mutation on plant heat response at the cellular level. We found that the *hts1* mutant displays significantly more ion leakage (1.8‐fold) than the wild‐type and *hts1*‐Com plants after heat treatment (Fig. 7a). However, ion leakage from the *hts1* mutant was comparable with the wild‐type under normal growth conditions, suggesting that the membrane fluidity is affected in the mutant due to heat stress. Transmission electron microscopy (TEM) showed more severe heat‐induced damage to the plasma membranes of the *hts1* mutant than those of the wild‐type (Fig. 7b). Confocal microscopy (Fig. 7c) and TEM (Fig. 7e) also revealed a higher occurrence of heat‐induced chloroplast deformation in the *hts1* mutant in contrast to the wild‐type and *hst1*‐Com1 leaves. After 2 h of heat treatment, more collapsed chloroplasts occurred in mesophyll cells of the mutant (60.5%) compared to the wild‐type (13.3%) and *hts1*‐Com1 (12.6%) (Fig. 7d).

### HTS1 is required for heat‐induced Ca^2+^ signaling and expression activation of *HsfA2* and its target genes

**Fig. 8:**
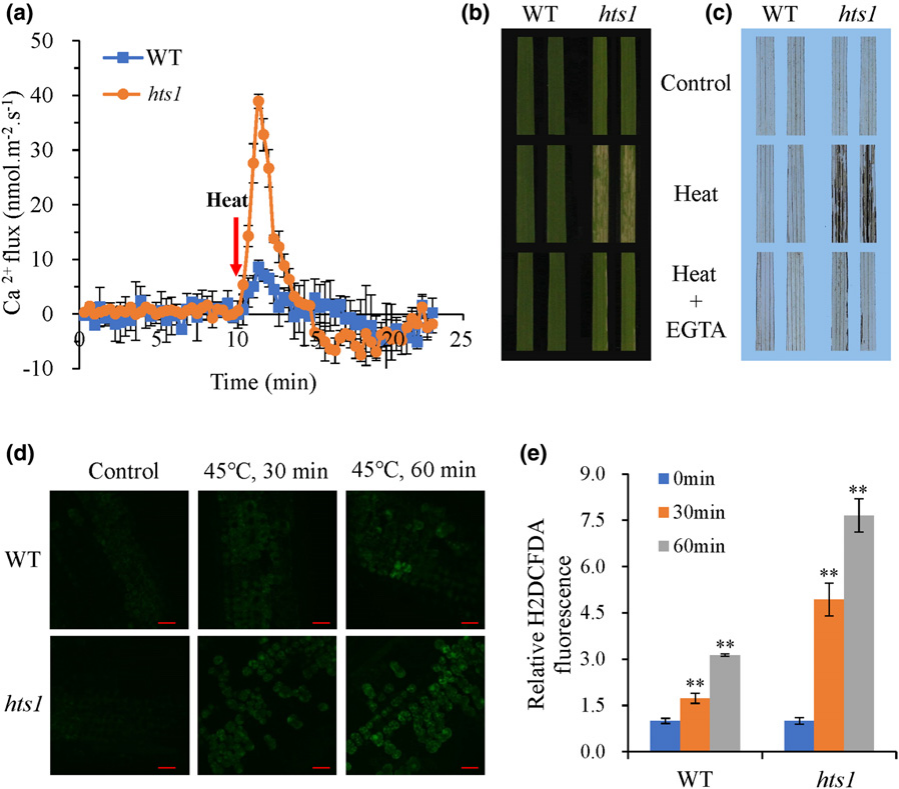
Intracellular Ca^2+^ influxes and H_2_O_2_ production are increased in the *hts1* mutant after heat treatment. (a) Transient net Ca^2+^ influxes measured from mesophyll cells of leaves of WT and the *hts1* mutant in response to heat treatment. Data are means ± SD (*n* = 5–8). (b) Heat‐challenged phenotypes of WT and the *hts1* mutant as examined with detached leaves with or without 100 μM EGTA supplement. (c) DAB staining of the heat‐challenged detached leaves of WT and *hts1* as described in (b). (d) Representative images of H2DCFDA fluorescence from mesophyll cells from leaves of WT and the *hts1* mutant measured in the control (28°C) and after 30 and 60 min of heat treatment (45°C). Bars, 15 μm. (e) Corrected total cell fluorescence of H2DCFDA for heat‐induced H_2_O_2_ production. Data are means ± SD (*n* = 5 with 30–50 cells). **, *P* < 0.01.

The plasma membrane is critical for plant heat sensing, cellular responses and Ca^2+^ signaling transduction (Niu & Xiang, 2018). Therefore, we monitored the heat stress‐induced Ca^2+^ influx in mesophyll cells using MIFEs. The heat‐induced, transient, net Ca^2+^ influx was much larger in mesophyll cells of the *hts1* mutant that, on average, was 4.1‐fold higher than in wild‐type cells during the first 10 min under heat treatment (Fig. 8a). Treatment with the Ca^2+^ chelator, EGTA, largely prevented heat‐induced H_2_O_2_ accumulation and leaf bleaching in detached leaves of the mutant (Fig. 8b,c). Confocal microscopy showed a gradual increase in fluorescence due to H_2_O_2_ in the mesophyll cells of the wild‐type and *hts1* mutant during the first 60 min of heat treatment. However, after 60 min, H_2_O_2_ production in *hts1* mesophyll cells was up to 7‐fold higher than in those of the control (Fig. 8d,e).

**Fig. 9:**
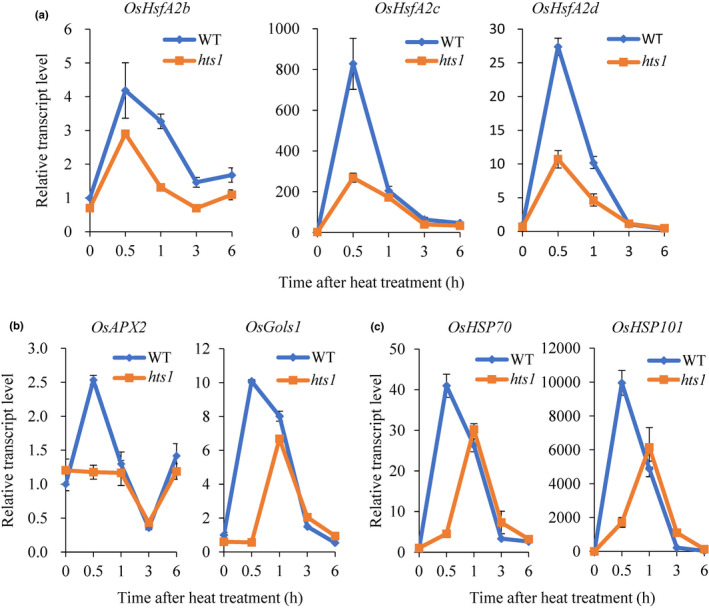
Mutation of *HTS1* inhibits transcriptional activation of *HsfA2* and its target genes in response to heat stress. qRT‐PCR analysis of mRNA levels of *HsfA2* (a), its representative target genes, including *APX2*, *GolS1* (b) and *HSP* genes (c) in leaves from 2‐wk‐old WT and mutant seedlings challenged with heat treatment (45°C) for the indicated times. The actin gene (*LOC_Os03g50885*) was used as the internal standard. Data represent means ± SD (*n* = 3).

As increasing intracellular Ca^2+^ and H_2_O_2_ levels indicate an induction of downstream, heat‐related, signaling pathways, we then examined the expression of HSF and HSP genes in response to heat treatment. Time course qPCR experiments showed that heat treatment leads to a sharp rise in the transcript levels of *HsfA2b*, *Hsf2Ac* and *HsfA2d* in wild‐type plants, reaching peaks after 30 min of treatment. However, the expression of *Hsf2Ac* and *HsfA2d* decreased to 32.4% and 39.1% of the wild‐type transcript levels, respectively (Fig. 9a). Compared with those of wild‐type plants, the heat‐induced expression of some *HsfA2* target genes (e.g. *APX2*, *GolS1*, *HSP70* and *HSP101*) were also markedly inhibited in the *hts1* mutant after 30 min of treatment (Fig. 9b,c). Moreover, the known heat tolerance‐related genes in rice, *OsNTL3*, *OsHATs*, *OsTOGR1* and *OsTT1*, showed different trends in the wild‐type and the mutant. *OsHATs* and *OsTOGR1* were significantly upregulated by heat treatment in the *hts1* mutant and differed significantly between the wild‐type and *hts1* mutant after 3 h (Fig. S12). Together, these results indicate that *HTS1* deficiency may cause rapid Ca^2+^ and ROS signaling that affects the transcriptional activation of *HsfA2s* and its target genes in response to heat stress in rice.

## Discussion

### *HTS1* encodes a functional KAR required for fatty acid synthesis and heat tolerance

Fatty acid‐based lipid synthesis and remolding are frequently linked to plant temperature stress responses, indicating the importance of targeting this metabolic pathway for the development of heat tolerance (Moellering *et al*., 2010; Wang *et al*., 2010; Higashi *et al*., 2015; Q. Li *et al*., 2015; Li *et al*., 2016; Barrero‐Sicilia *et al*., 2017; Higashi *et al*., 2018; Niu & Xiang, 2018). It was reported that high temperature causes a decline of KAS abundance and impairing fatty acid accumulation in *fabG* (Ts) and *envM* mutants that results in a heat‐induced, lethal phenotype in bacteria such as *E. coli* (Bergler *et al*., 1994; Lai & Cronan, 2004). It was also found that the Arabidopsis *mod1* mutant with an ENR activity deficiency affecting the fatty acid synthase complex that catalyzes *de novo* synthesis of fatty acids has increased sensitivity to high temperature (Mou *et al*., 2000). In the present study, we identified and cloned *HTS1*, which encodes a functional KAR protein in rice. Rice plants lacking *HTS1* had reduced fatty acid accumulation and exhibited a heat‐sensitive phenotype when treated at 45°C. HTS1 contains a chloroplast transit sequence and its targeting to thylakoids was confirmed experimentally (Fig. 4), validating *de novo* fatty acid synthesis in chloroplasts (Troncoso‐Ponce *et al*., 2016). We showed that the *hts1* mutant accumulates higher contents of H_2_O_2_ and MDA and has greater levels of cell death at high temperature compared with the wild‐type (Figs 1, S4). These physiological traits were associated with significantly lower fatty acid contents (Table 1), suggesting an important role of *HTS1* in fatty acid biosynthesis and heat stress adaptation of rice plants.

Sequence analysis revealed that the *hts1* mutant carries a single base transition (G to A) that changes an Ala to a Thr in the coding sequence of *HTS1* (Fig. 2). This amino acid substitution causes a large decrease in KAR activity, leading to a 19.6% reduction in the total fatty acid content of *hts1* plants relative to the wild‐type (Fig. 5; Table 1). This moderate reduction of fatty acids in *hts1* is consistent with data for the *Arabidopsis ENR* or *KAS1* knockout mutants, which also maintained sufficient fatty acids for survival due to low residual enzyme activities (Mou *et al*., 2000; Wu & Xue, 2010), indicating that plant fatty acid biosynthesis is robust and can be maintained even in the face of genetic lesions in the primary synthesis steps (Li, Q *et al*., 2015). However, unlike the *Arabidopsis mod1* and *ksa1* mutants, which showed more severe morphological changes, the *hts1* mutant only showed slight growth inhibition under normal growth conditions (Figs S1, S2). This may be due to genetic redundancy, because there are two putative KAR homologs in the rice genome (Fig. S7). We found that the HTS1 homolog (*LOC_Os02g30060*) is also located in chloroplasts and its expression is significantly higher in the *hts1* mutant (Fig. S13), suggesting a potential redundant role with HTS1.

The fatty acid metabolism pathway and the core enzymes for fatty acid biosynthesis are conserved throughout evolution due to their essential metabolic roles in living organisms (Campbell & Cronan, 2001; Kohli *et al*., 2016). KAR (HTS1) is one of the type II FAS enzymes that were transferred from the plastid to the nuclear genome during their evolutionary history (Kohli *et al*., 2016). Bioinformatic analysis showed that HTS1 homologs ubiquitously exist in land plant and algal species, ranging from the streptophyte algae to angiosperm plants. The conserved KR domain of *Klebsormidium flaccidum* (a charophyte alga) is more similar to those in other land plant species including *Oryza sativa* compared to those in *Volvox carteri* (a chlorophyte alga) and *Cyanidioschyzon merolae* (a rhodophyte alga) (Fig. 3c). Thus, we suggest that HTS1 proteins are highly conserved in land plants (Figs 3b, S7, S8; Table S2). There may be an early evolutionary origin of these nuclear‐encoded proteins and the protein precursors that are targeted into plastids using an N‐terminal chloroplast transit peptide (Bruce, 2000), which is an interesting topic for further study. Given the important roles of fatty acids and their derivatives in response to major abiotic stresses including cold, heat, drought and salt (Liu *et al*., 2019; He & Ding, 2020), and the role of HTS1 in the response of rice to heat, we propose that the origin of HTS1s in streptophyte algae may be important in the successful evolutionary transition from aquatic to terrestrial living of green plants (Zhao *et al*., 2019; Feng *et al*., 2020).

### *HTS1* confers heat tolerance via modulating membrane lipid stability

Plant cells surviving under extreme temperatures need to first maintain lipid membrane stability and fluidity by regulating lipid saturation (Niu & Xiang, 2018). It has been reported that heat stress significantly affects membrane lipid composition and thus increases saturation of membrane lipids, enhancing ROS production and membrane lipid peroxidation, resulting in membrane disruption and cell damage (Wahid *et al*., 2007; Balogh *et al*., 2013). Recent studies on the lipidome in Arabidopsis revealed that heat stress specifically decreases the levels of 16 : 3‐ and/or 18 : 3‐containing chloroplastic glycerolipids (e.g. MGDG) and increases the production PC, acyl‐MGDG, TAG, and digalactosyldiacylglycerol in leaves (Higashi *et al*., 2015; Higashi & Saito, 2019). Research using the green alga *Dunaliella bardawil* also suggests that the accumulation of polyunsaturated membrane lipids converted from DG are important in the reduction of heat damage to cell membranes (Liang *et al*., 2020). Interestingly, the *hts1* mutant had significantly reduced contents of C16 and C18 fatty acids, resulting in low accumulation of glycerolipids, such as MGDG and DG, the major structural constituents of cellular membranes rich in polyunsaturated acids (Fig. 6). Therefore, it is not surprising that the lower fatty acid contents in *hts1* mutant plants significantly weakened their membranes, especially during heat stress, aggravating the oxidation of membrane lipids in mutant cells and leading to severe cell membrane disruption and plastid degradation (Figs 1d–f, 7). In addition, previous studies showed that heat‐induced lipid remodeling depends on the rate of *de novo* fatty acid synthesis and the rate of turnover of membrane lipids (Bao *et al*., 2000; Falcone *et al*., 2004). Here, the upregulation of HTS1 transcripts and protein under high temperature (Fig. 4c,d) and a high level of thermal stability of HTS1 (Figs 5d,e, S11) suggest that HTS1‐mediated fatty acid biosynthesis is required for lipid metabolism under heat stress. Together, these results highlight that the resistance of plants to heat stress depends on abundant fatty acid synthesis for maintaining membrane stability.

### Potential role of ROS, Ca^2+^ and HSF in HTS1‐mediated heat stress response in rice

Heat stress causes ROS accumulation in organelles, which is an indispensable factor for evoking the HSR in plants, but an excessive heat‐induced elevation of ROS production leads to oxidative stress and cell death (Suzuki *et al*., 2012). Chloroplastic and mitochondrial metabolism and plasma membrane NADPH oxidases are the most prominent sources of H_2_O_2_ production under heat stress (Suzuki *et al*., 2012; Sun & Guo, 2016; Choudhury *et al*., 2017). During heat treatment, the fatty acid‐deficient *hts1* mutant significantly accelerated H_2_O_2_ accumulation (Figs 1e, 8d,e) and cell death compared with the wild‐type (Fig. 1d). It was revealed that a deficiency in fatty acid biosynthesis in the Arabidopsis *mod1* mutant causes cell death by modulating ROS generation through impairment of the mitochondrial electron transport chain (Mou *et al*., 2000; Wu *et al*., 2015; Zhao *et al*., 2018, 2020). Therefore, we suggest that H_2_O_2_ overproduction is a possible explanation for the heat‐sensitive phenotype of the *hts1* mutant. Moreover, ROS‐scavenging enzymes such as APX and CAT are heat stress‐inducible (Ohama *et al*., 2017). We also found that heat treatment significantly upregulated APX and CAT enzyme activities in wild‐type plants, but downregulated them in the *hts1* mutant (Fig. S4) and an H_2_O_2_ scavenger, GSH, partially rescued the heat‐sensitive phenotype in the *hts1* mutant (Fig. S5). Thus, the excessive H_2_O_2_ accumulation in the *hts1* mutant may be a combined effect of the increased heat‐induced H_2_O_2_ generation and the decreased H_2_O_2_ scavenging capacity.

The plasma membrane acts as the first responder to abiotic stresses, including heat stress (Barkla & Pantoja, 2011). High temperature affects the fluidity and stability of plasma membranes activating membrane‐associated heat sensors, including calcium channels, resulting in a rapid Ca^2+^ influx that, in turn, triggers a signal transduction cascade (Finka *et al*., 2012; Mittler *et al*., 2012; Zhao *et al*., 2021). It was shown that a change of membrane lipid composition largely affects heat sensing and modulates Ca^2+^‐dependent heat shock responses in plants (Saidi *et al*., 2010). Here, we detected a larger rapid, transient increase in Ca^2+^ influx in mesophyll cells of the *hts1* mutant during the first 10 min of heat treatment than that of the wild‐type (Fig. 8a), and using a Ca^2+^ chelator, EGTA, can reduce the heat‐induced H_2_O_2_ production and cell death in leaves of the *hts1* mutant (Fig. 8b). This suggested that Ca^2+^ may be involved in the *HTS1*‐mediated plant HSR, but the link between Ca^2+^ signaling and the function of HTS1 requires further investigation.

Moreover, heat stress induces rapid accumulation of H_2_O_2_ and a rise of cytosolic Ca^2+^, which are involved in HSF activation (Volkov *et al*., 2006; Ohama *et al*., 2017). Interestingly, we found that the expression of *HsfA2c* and *HsfA2d* are significantly upregulated in wild‐type plants (Fig. 9a), indicating that the Ca^2+^‐mediated *HsfA2* signaling pathway may be a significant mechanism associated with heat resistance in rice. *HsfA2* is considered as a direct target of *HsfA1* and is essential for the activation of HSR in plants (Charng *et al*., 2007). The activation of *HsfA2* can further modulate the expression of many downstream target genes, including HSPs and other HS‐induced genes (Figs 9b,c) that increase tolerance to heat stress (Ohama *et al*., 2017). Here, a high Ca^2+^ influx in mesophyll cells under heat stress was associated with a marked suppression of the heat‐induced upregulation of *HsfA2* and its target genes in the *hts1* mutant (Figs 8a, 9). These findings support an important role for *HsfA2* in *HTS1*‐mediated HSR.

### Conclusions

**Fig. 10:**
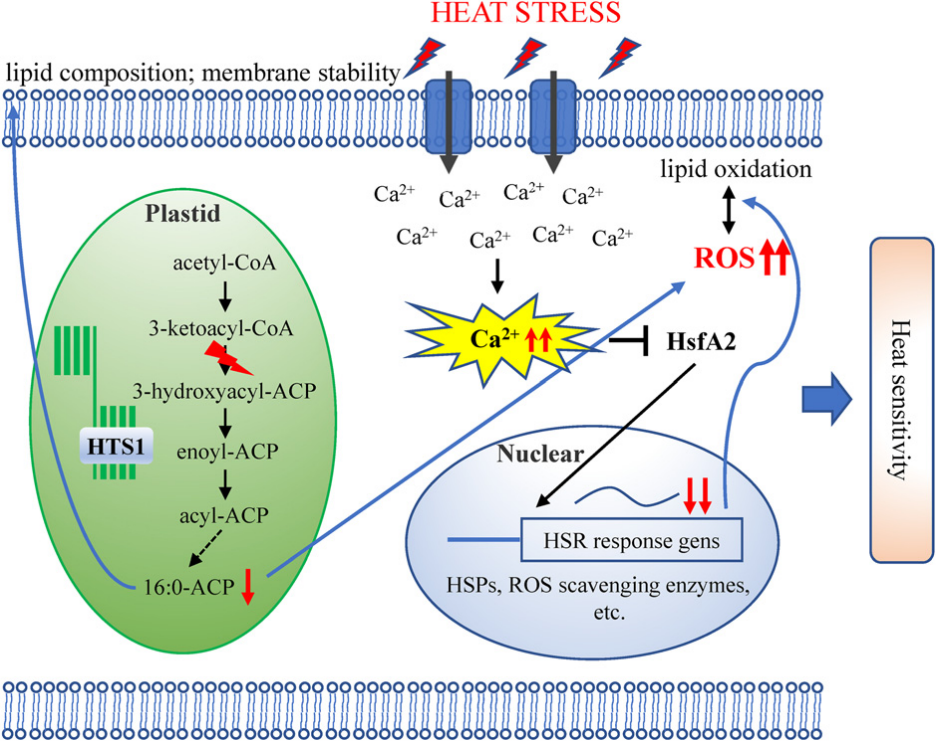
A proposed working model of HTS1 in modulating heat tolerance in rice (*Oryza sativa*). A deficiency of HTS1 directly impairs the biosynthesis of fatty acids in the *hts1* mutant. The reduced fatty acid content (mainly C16 : 0 and C18 : 3) disrupts the integrity and stability of cell membrane systems under heat stress, which may cause abnormal heat‐induced reactive oxygen species (ROS) and Ca^2+^ signaling. The mutation in *HTS1* also causes a repression of the transcriptional activity of heat stress transcription factor (HSF) and heat stress protein (HSP) networks, leading to an impaired heat stress response (HSR) regulation and uncontrolled heat damage. Red arrows represent increased/decreased expression or content. Dashed arrow indicates multiple steps. Blunt‐ended arrow indicates inhibition.

We conclude that HTS1 deficiency directly impairs fatty acid biosynthesis and lipid metabolism in the *hts1* mutant. The reduced fatty acid content (mainly C16 : 0 and C18 : 3) disrupts the integrity and stability of cell membrane systems under heat stress, causing abnormal heat‐induced Ca^2+^ and ROS signal transduction cascades. The mutation in HTS1 also causes a repression of the transcriptional activity of HSF and HSP networks, leading to an impaired HSR regulation and uncontrolled heat damage. Therefore, we propose that HTS1 is a key factor for *de novo* fatty acids synthesis and is essential for heat tolerance in rice through effects on membrane stability, heat stress‐related transcriptional activity, and potentially Ca^2+^ and ROS signaling (Fig. 10). Insights into the relationship of fatty acid synthesis and stress signaling under heat stress will be beneficial for a better understanding towards breeding plants for future climate conditions.

## Author contributions

YY, Z‐HC, LW and QQ conceived and supervised the project; FC, GD, FW, YS, JZ, YZ, BR, YW, XF, CZ and MTY conducted the experiments; FC, DZ, Z‐HC and YY designed the experiments and analyzed the data; FC, PH, Z‐HC and YY wrote the manuscript. All authors read and approved the final manuscript. FC and GD contributed equally to this work.

## Supporting information


**Fig. S1** The *hts1* mutant exhibits a pale green phenotype.
**Fig. S2** Phenotypic characterization of wild‐type and *hts1* mutant rice (*Oryza sativa*).
**Fig. S3** HTS1 is not a general factor response for environmental stress tolerance.
**Fig. S4** HTS1 is involved in the heat‐induced oxidative stress response.
**Fig. S5** Application of exogenous glutathione partially reverses the heat‐sensitive phenotype of the *hts1* mutant.
**Fig. S6** Growth recovery of *hts1*‐Com plants at the seedling and reproductive stages under field conditions.
**Fig. S7** Phylogenetic trees and sequence alignment of HTS1 and its homologous proteins in plants.
**Fig. S8** Phylogenetic analysis of HTS1 in different plant and algal species selected from the 1KP database.
**Fig. S9** Anti‐HTS1 antibody detection by western blotting.
**Fig. S10** Expression patterns of *HTS1* in rice (*Oryza sativa*).
**Fig. S11** Western blot of HTS1 protein incubated at 25°C (control) or 45°C for the indicated time.
**Fig. S12** Expression and subcellular localization of two rice KAR homologs.
**Fig. S13** Expression levels of rice heat‐resistance‐associated genes in *hts1* and WT plants under heat treatment.

**Table S1** List of primers used in this study.
**Table S2** Statistics of evolution of HTS1s in the 1KP dataset.
**Table S3** The proportions of identified lipid classes in leaves of 2‐wk‐old rice wild‐type and *hts1* mutant seedlings.
**Table S4** All detected lipid species in leaves of 2‐wk‐old rice wild‐type and *hts1* mutant seedlings.
Please note: Wiley Blackwell are not responsible for the content or functionality of any Supporting Information supplied by the authors. Any queries (other than missing material) should be directed to the *New Phytologist* Central Office.
