# Supplementary material for: A β‐ketoacyl carrier protein reductase confers heat tolerance via the regulation of fatty acid biosynthesis and stress signaling in rice
Source: New Phytol. 2021 Jul 30;232(2):655–72. doi: 10.1111/nph.17619 (PMC9292003; doi:10.1111/nph.17619)
Supplement: Supplementary file 1 — Fig. S1 The hts1 mutant exhibits a pale green phenotype. Fig. S2 Phenotypic characterization of wild‐type and hts1 mutant rice (Oryza sativa). Fig. S3 HTS1 is not a general factor response for environmental stress tolerance. Fig. S4 HTS1 is involved in the heat‐induced oxidative stress response. Fig. S5 Application of exogenous glutathione partially reverses the heat‐sensitive phenotype of the hts1 mutant. Fig. S6 Growth recovery of hts1‐Com plants at the seedling and reproductive stages under field conditions. Fig. S7 Phylogenetic trees and sequence alignment of HTS1 and its homologous proteins in plants. Fig. S8 Phylogenetic analysis of HTS1 in different plant and algal species selected from the 1KP database. Fig. S9 Anti‐HTS1 antibody detection by western blotting. Fig. S10 Expression patterns of HTS1 in rice (Oryza sativa). Fig. S11 Western blot of HTS1 protein incubated at 25°C (control) or 45°C for the indicated time. Fig. S12 Expression and subcellular localization of two rice KAR homologs. Fig. S13 Expression levels of rice heat‐resistance‐associated genes in hts1 and WT plants under heat treatment. [file NPH-232-655-s002.pdf]

A β-Ketoacyl Carrier Protein Reductase Confers Heat Tolerance via the Regulation of Fatty Acid Biosynthesis and Stress Signaling in Rice

Fei Chen[1†], Guojun Dong[2†], Fang Wang[3], Yingqi Shi[1], Jiayu Zhu[1], Yanli Zhang[1], Banpu Ruan[1], Yepin Wu[1], Xue Feng[4], Chenchen Zhao[5], Miing T. Yong[5], Paul Holford[5], Dali Zeng[2], Qian Qian[2], Limin Wu[1*], Zhong-Hua Chen[5,6*], Yanchun Yu[1*]

[1]College of Life and Environmental Sciences, Hangzhou Normal University, Hangzhou 311121, China

[2]State Key Laboratory for Rice Biology, China National Rice Research Institute, Hangzhou 310006, China

[3]Institute of Insect Sciences, Zhejiang University, Hangzhou 310058, China

[4]College of Agronomy, Qingdao Agricultural University, Qingdao 266109, China

[5]School of Science, Western Sydney University, Penrith, NSW 2751, Australia

[6]Hawkesbury Institute for the Environment, Western Sydney University, Penrith, NSW 2751, Australia

[†]These authors contributed equally to this paper.

[*]Author for correspondence:

Limin Wu, Email: lmwu2006@aliyun.com; Zhong-Hua Chen, Email: z.chen@westernsydney.edu.au; Yanchun Yu, Email: ycyu@hznu.edu.cn

Article acceptance date: 5 July 2021

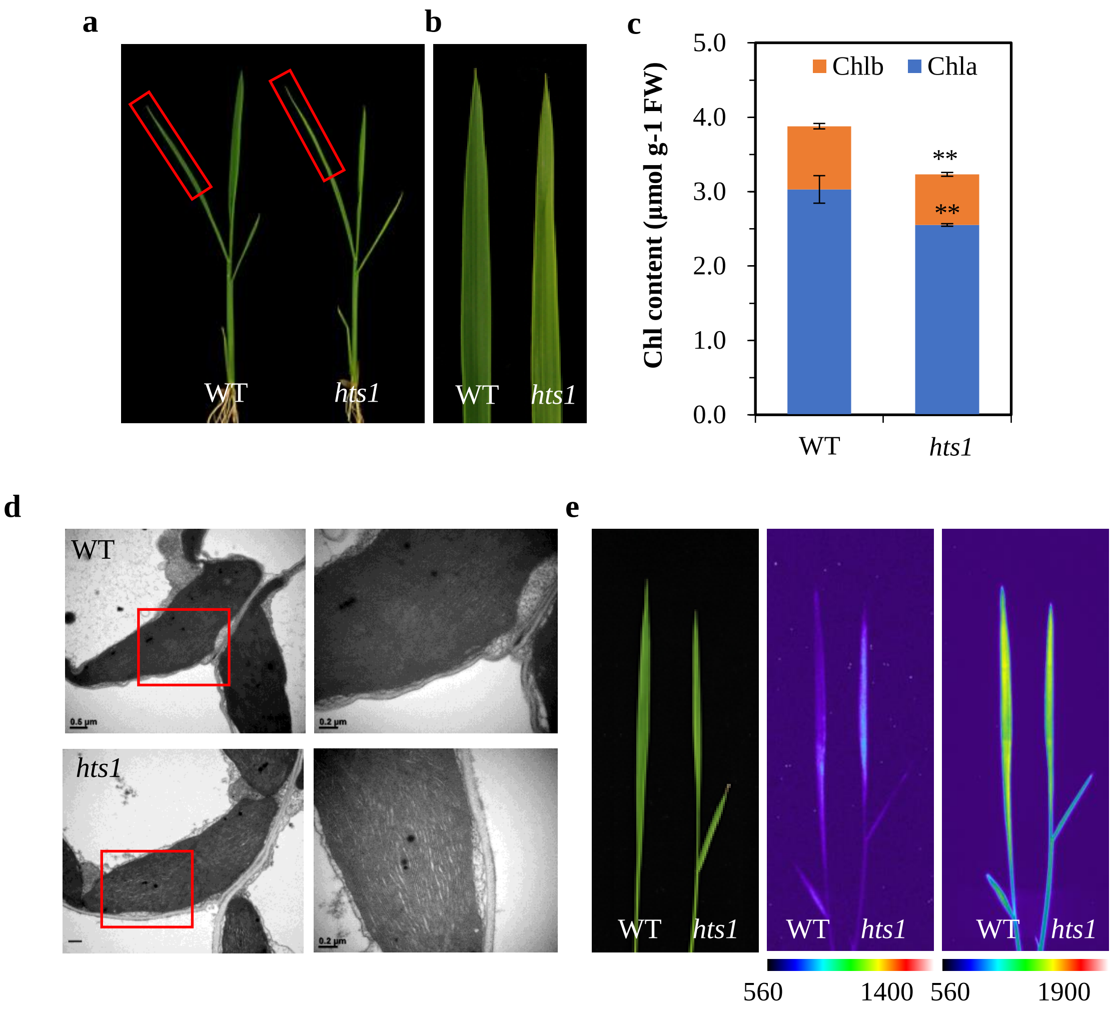

Fig. S1. The *hts1* mutant exhibits a pale green phenotype. (a) Phenotypes of 2-week-old wild-type (WT) and *hts1* seedlings grown in solution culture. (b) Enlarged views of leaves from (a). (c) Chlorophyll contents of WT and *hts1* mutant leaves two weeks after germination. Data are means ± SD (n = 5). ** $p < 0.01$. Chla, chlorophyll a; Chlb, chlorophyll b; FW, fresh weight. (d) Transmission electron microscopy of chloroplasts from the first fully expanded leaves of 2-week-old seedlings of the WT and *hts1* mutant. Scale bars: 1.0 μm (left) and 0.5 μm (right). (e) Chlorophyll autofluorescence of the WT and *hts1* mutant. Leaves from 7-day-old seedlings were collected for observation. Left, bright field images; middle, chlorophyll fluorescence (*Fo*) of plant in the dark; right, fluorescence after 2 min illumination (*Fs*). The pseudocolor bar below shows the range of luminescence intensity in each image.

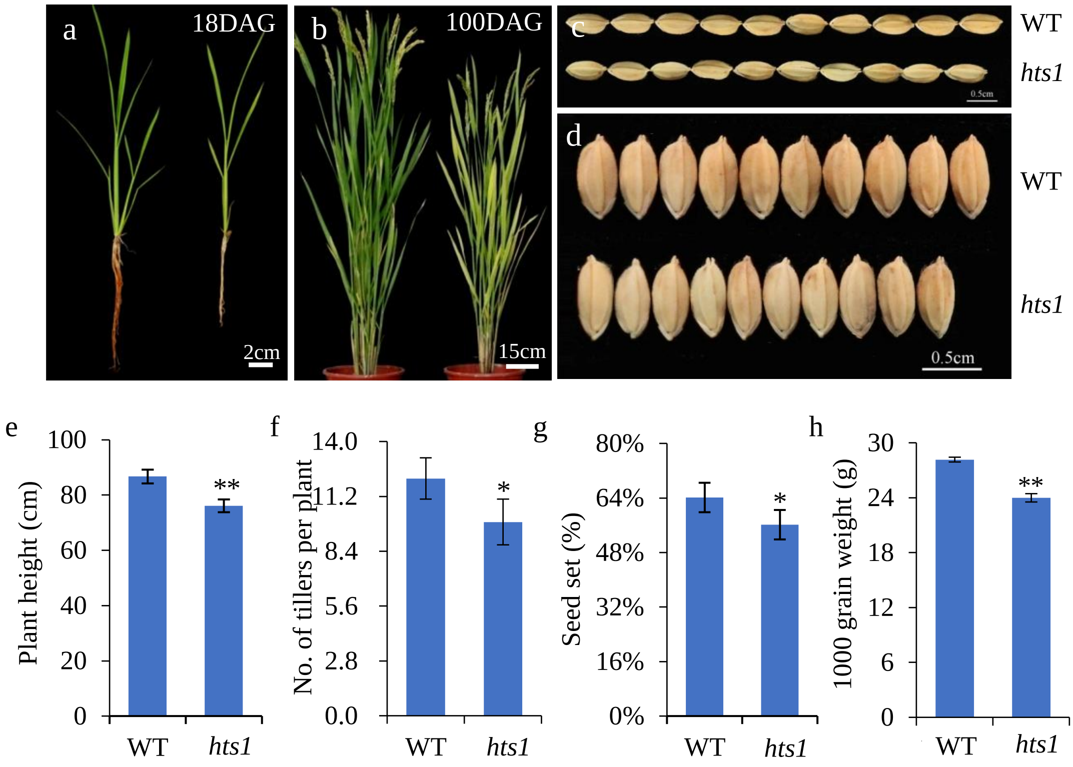

Fig. S2. Phenotypic characterization of wild-type (WT) and *hts1* mutant rice (*Oryza sativa*). (a, b) Plant growth 18 and 100 days after germination (DAG). Right, WT; left, *hts1*. (c, d) The seed size phenotype of WT and *hs1* mutant. (e-h). Quantitation of agronomic traits in the WT and *hts1* mutant. Data are means ± SD (n=10). * P < 0.05 and **. P < 0.01

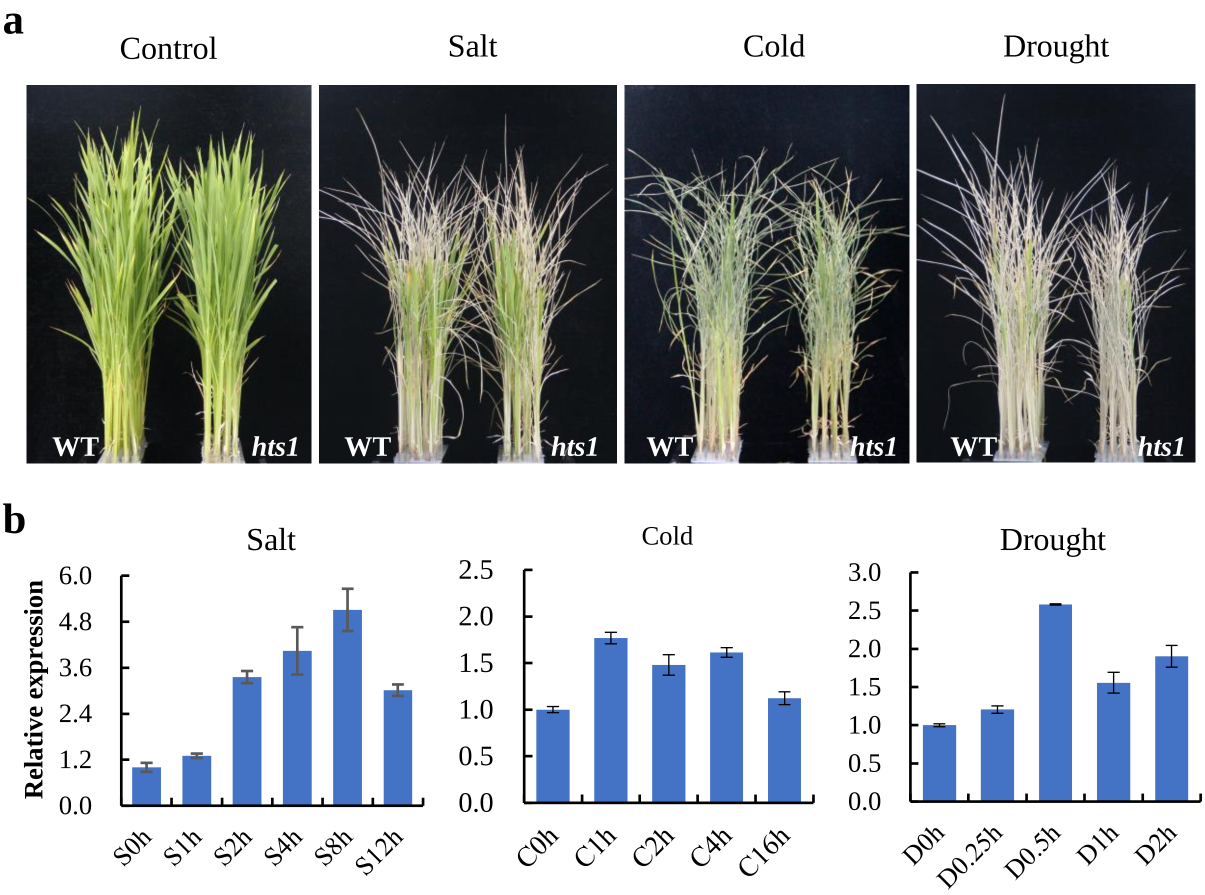

Fig. S3. *HTS1* is not a general response factor for environmental stress tolerance. (a) Growth characterization of wild type (WT) and *hts1* mutant seedlings under salt, cold or drought stress. Two-week-old WT and *hts1* mutant seedlings were treated with cold stress (exposing plants to 4 °C), salt stress (10g/L NaCl), or drought stress (20% of polyethylene glycol-6000 (PEG)), respectively. (b) Expression levels of *HTS1* following short-term abiotic stresses. Data are means ± SD (n = 3).

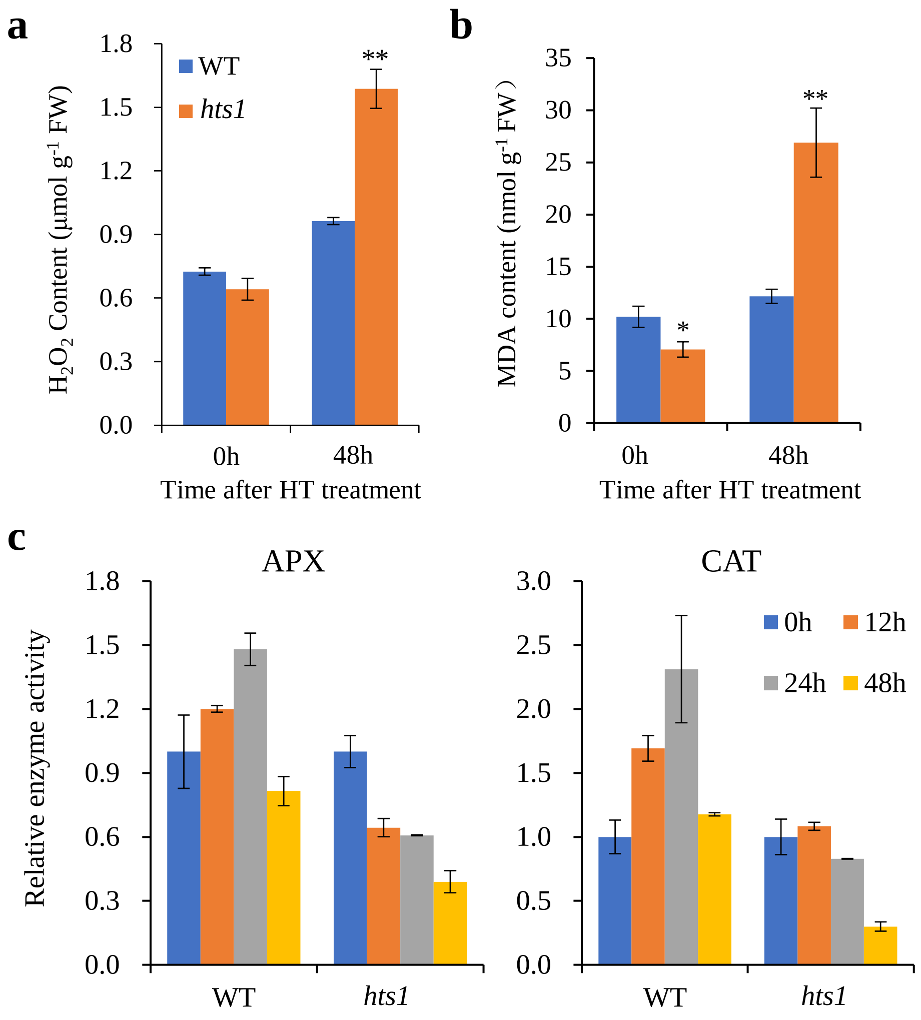

Fig. S4. *HTS1* is involved in heat-induced oxidative stress responses. (a, b) Determination of H_2O_2 and MDA content in leaves of 2-week-old WT and *hts1* mutant seedlings grown at 28 °C and treated with heat (45 °C) for 0 and 48 h. (c) APX and CAT activities of 2-week-old WT and *hts1* seedlings grown at 28 °C then treated at 45 °C for 0, 12, 24 and 48 h. Data are means ± SD (n = 3). * $P < 0.05$, ** $P < 0.01$.

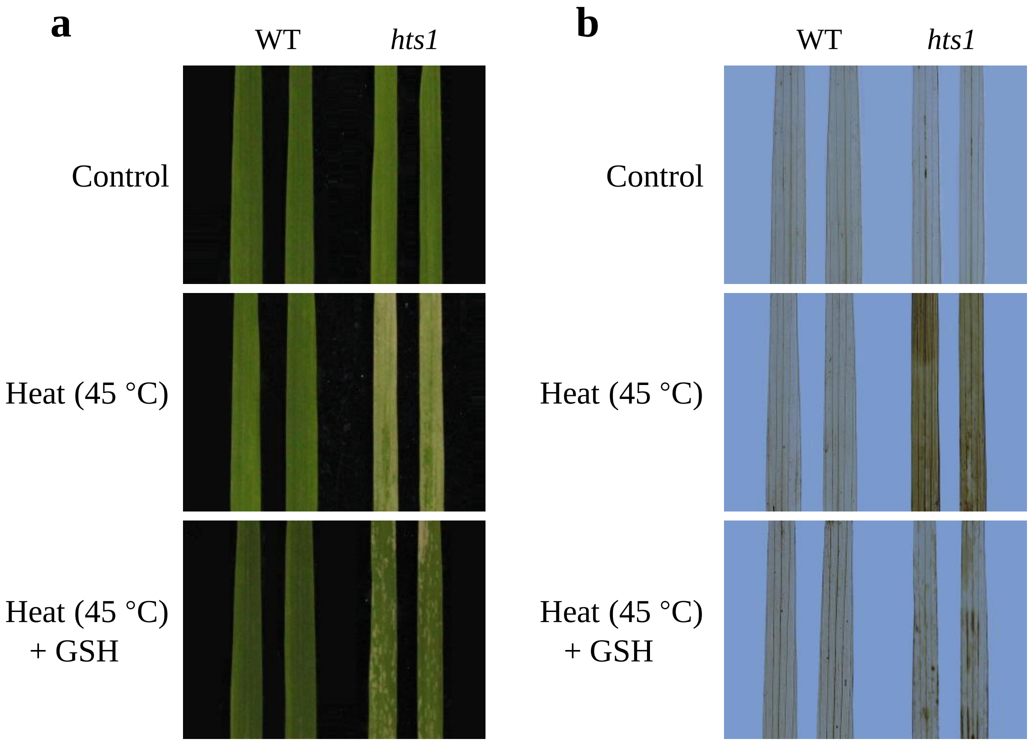

Fig. S5. Application of exogenous glutathione partially reverses the heat sensitive phenotype of *hts1* mutant. (a) Heat-challenged phenotypes of WT and *hts1* mutant as examined with detached leaves with or without external glutathione supplement. (b) DAB staining of the heat-challenged detached leaves of the WT and *hts1* mutant as described in (a).

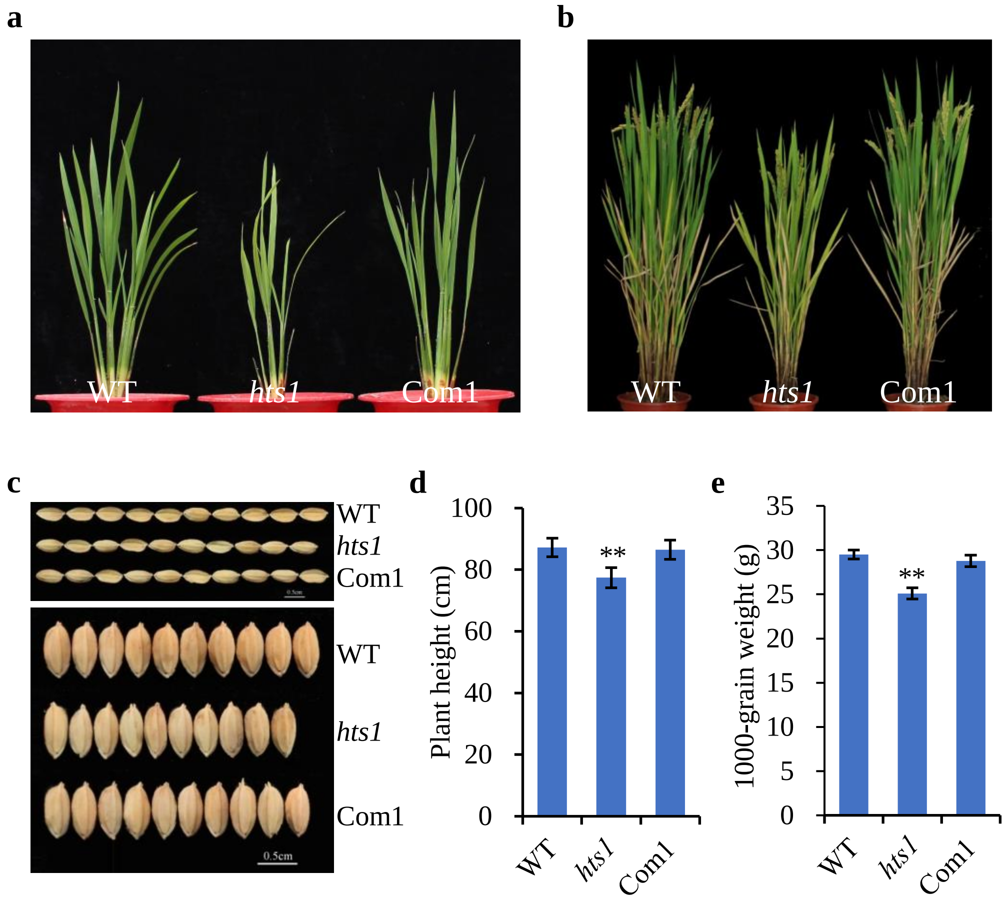

Fig. S6. Growth recovery of *hts1*-Com plants at the seedling and reproductive stages in field condition. (a, b) Gross morphology of wild-type (WT), *hts1* and *hts1*-Com (Com1) plants at seedling (a) and reproductive (b) stages. (c) The seed size phenotype of WT, *hts1* and Com1 plants . (d, e). Statistical data of the plant height (d) and 1000-grian weight (e) in WT, *hts1* and Com1 plants. Data are means ± SD (n=10). ** P < 0.01.

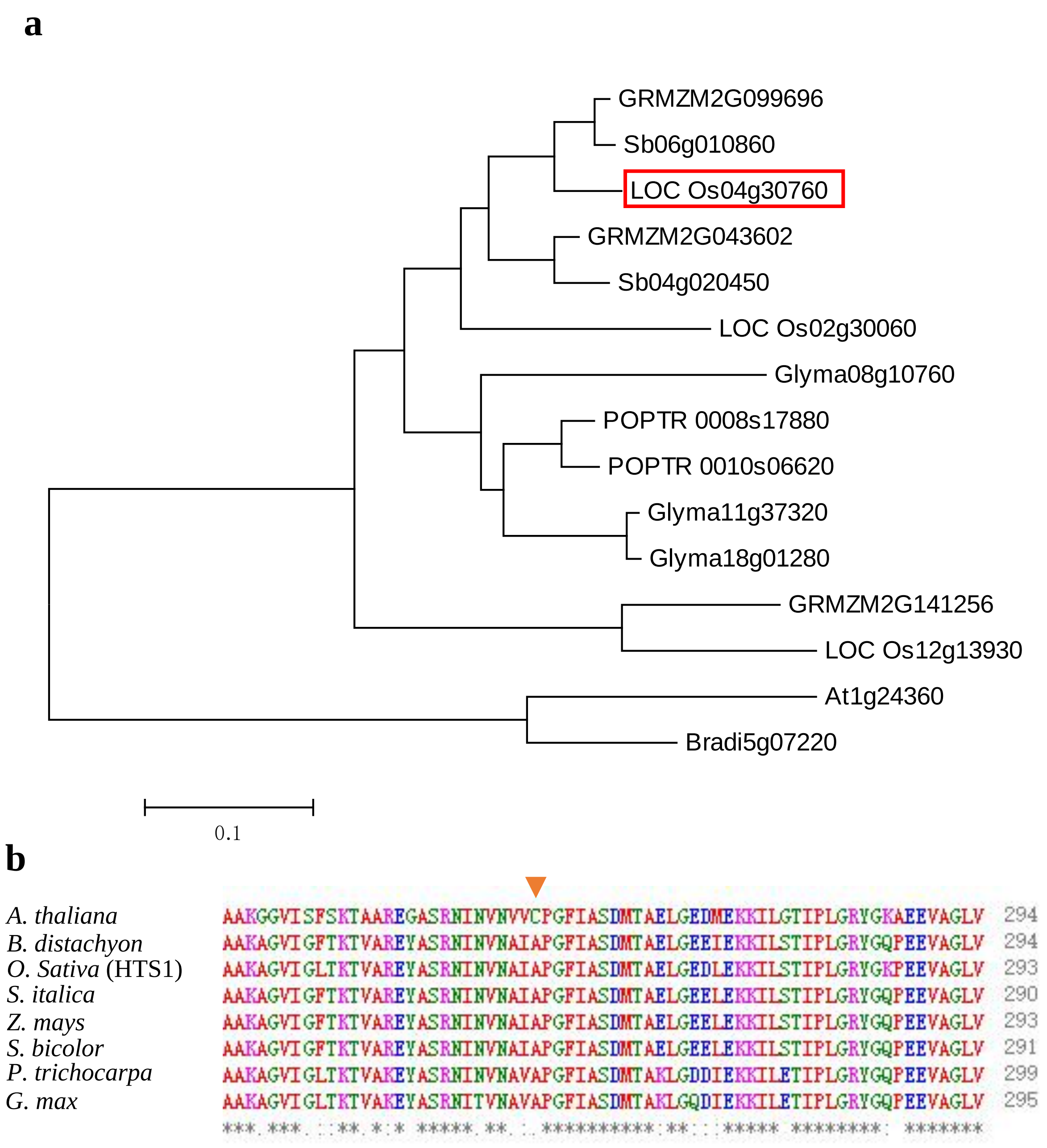

Fig. S7. Phylogenetic analysis and sequence alignment of HTS1 and its homologous proteins in plants. (a) Phylogenetic tress of HTS1 in plants. Amino acid sequences of HTS1 orthologous proteins were analyzed using MEGA5 software (Version 5.05) using maximum likelihood. The numbers at the nodes represent percentage bootstrap values based on 1,000 replications. HTS1 (LOC_Os04g30760) is highlighted by the red box. (b) Sequence alignment of HTS1 and its homologous. The symbols below each position in the sequence indicate the amount of conservation ('*' exact; ':' conserved substitution; '.' semi-conserved substitution). The red arrowhead represents the mutation site of A to T in the *hts1* mutant. HTS1 proteins from *A. thaliana*, *B. distachyon*, *O. Sativa*, *S. italica*, Z. mays, *S. bicolor*, *P. trichocarpa* and *G. max* are analyzed.

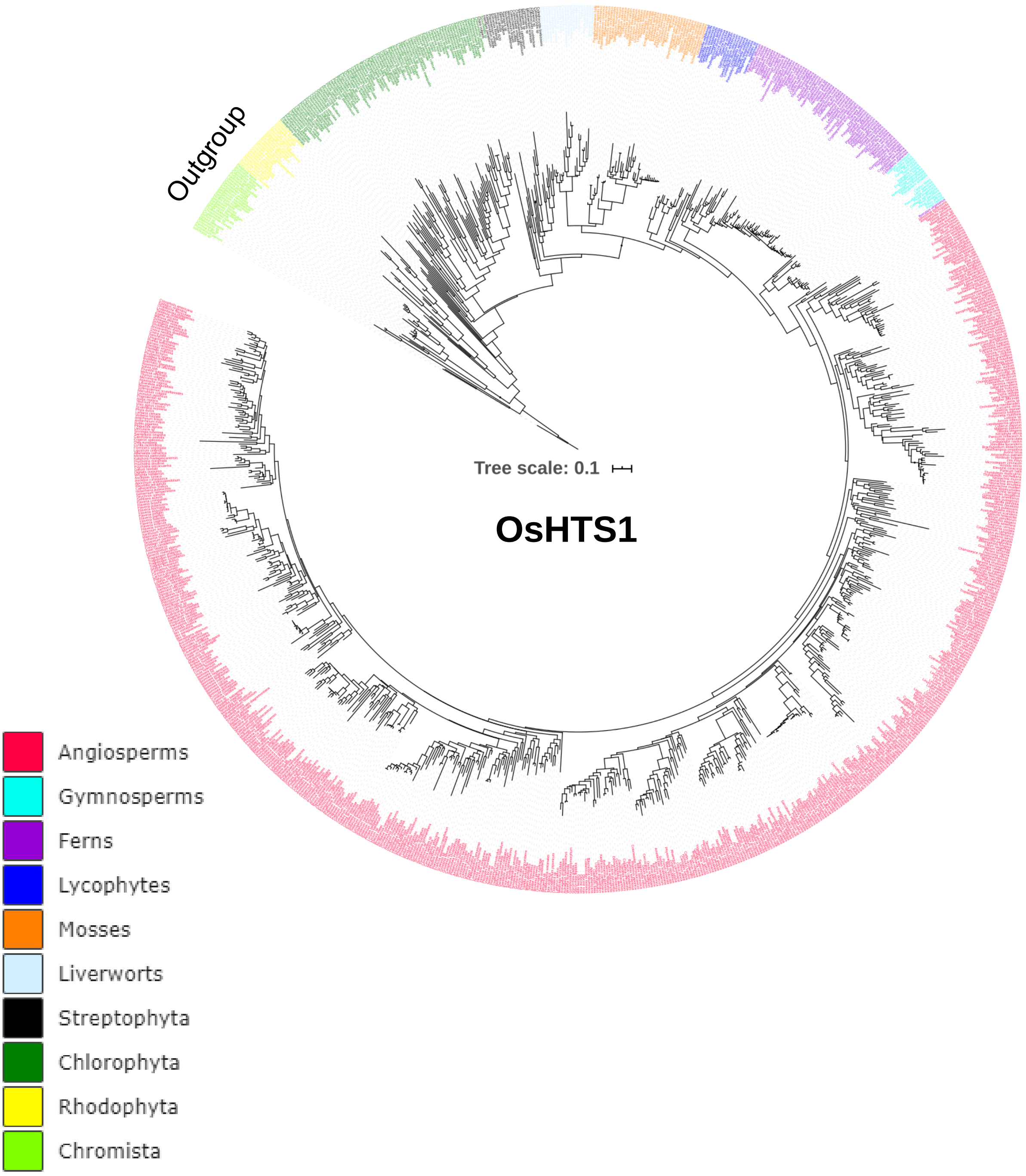

Fig. S8. Phylogenetic analysis of HTS1 from different plant and algal species selected from the 1KP database. All the transcriptomes can be accessed from the One Thousand Plants Project. Candidate protein sequences were acquired by BLASTP searches using OsHTS1 as the query with the criterion of E-value<10^{-5}.

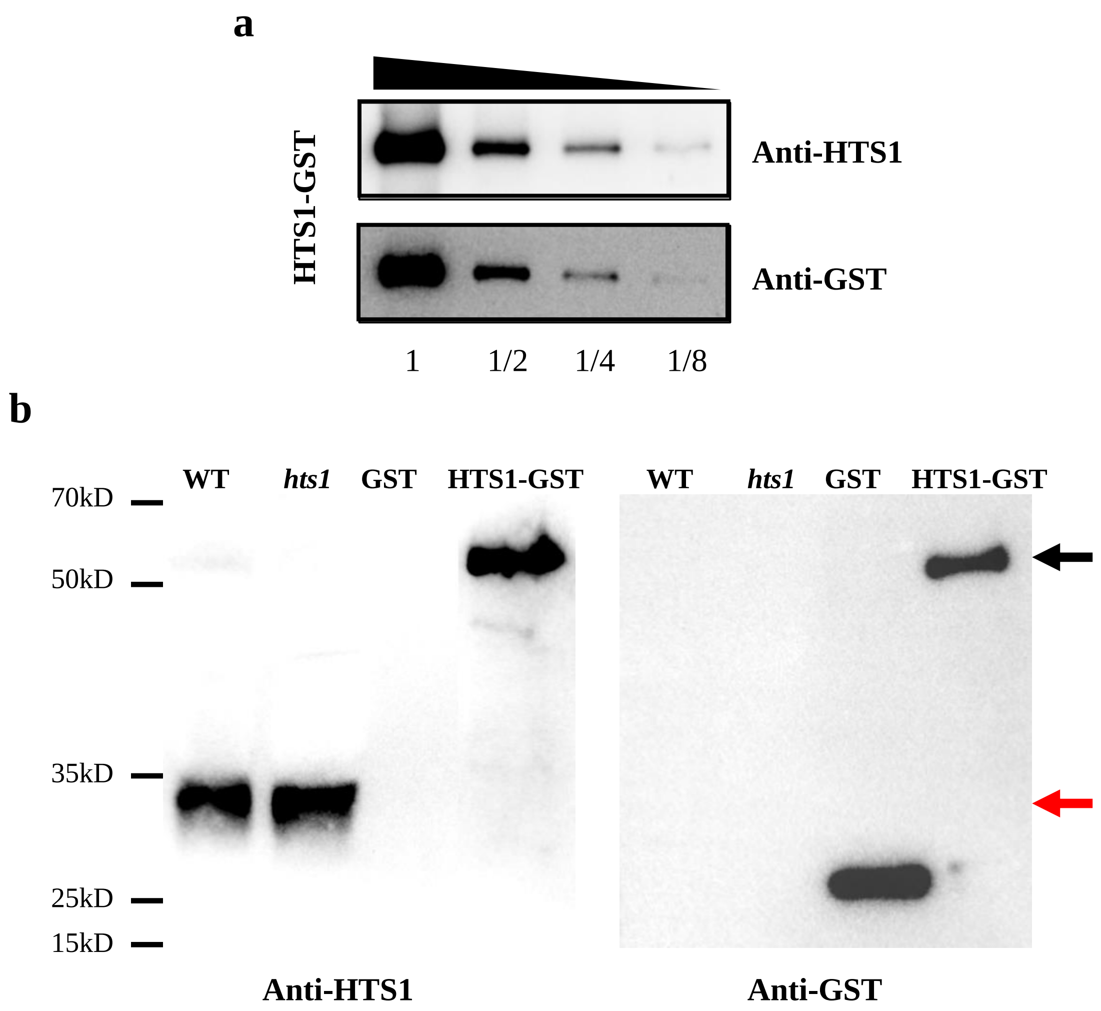

Fig. S9. Anti-HTS1 antibody detection by western blotting. (a) Western blot analysis of dilution series of purified HTS1-GST protein using anti-HTS1 and anti-GST antibodies. (b) HTS1 antibodies specifically detect the endogenous HTS1 protein and the HTS1-GST fusion protein. Red and black arrows indicate the endogenous HTS1 protein and the HTS1-GST fusion protein respectively. WT, wild type.

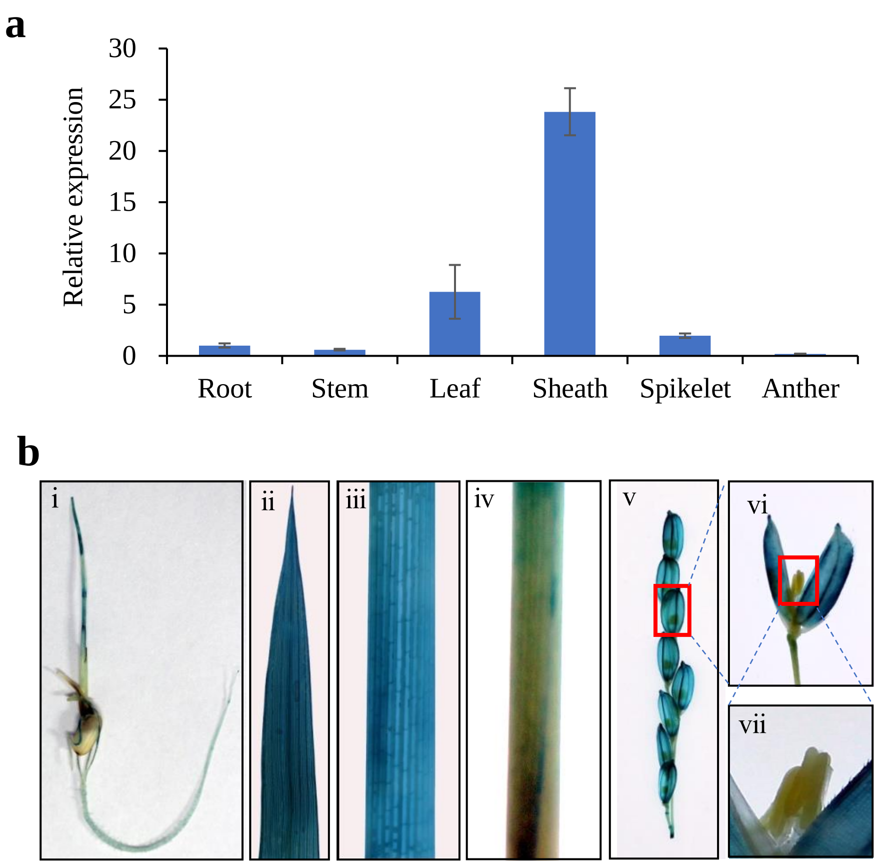

Fig. S10. Expression patterns of *HTS1* in rice (*Oryza sativa*). (a) qRT-PCR analysis of *HTS1* expression in organs, including roots, stems, leaves, leaf sheaths, spikelet and anthers. The Actin gene (*LOC_Os03g50885*) was used as an internal control. Data represent means ± SD (n = 3). (b) *HTS1* expression patterns revealed by promoter-GUS fusion analysis in transgenic plants. GUS activity was observed in the young seedling (i), leaf (ii), sheath (iii), stem (iv), spikelet (v), glume (vi), and anthers (vii).

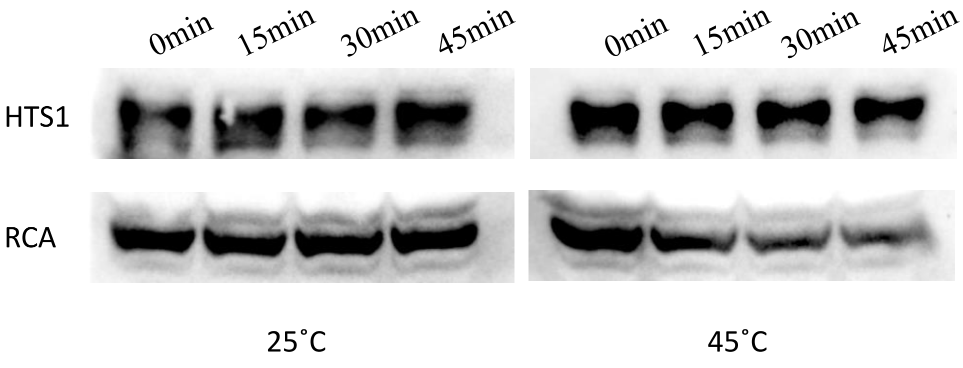

Fig. S11. Western blot of HTS1 protein incubated at 25 °C (control) or 45 °C for the indicated times. Total proteins were extracted from leaves of 2-week-old wild-type seedling and incubated at 25 °C (control) or 45 °C for the indicated time. Following incubation, samples were centrifuged and the supernatant of each sample was analyzed. Equal volumes of protein samples were loaded. Rubisco activase alone (RCA) protein was used as the negative control.

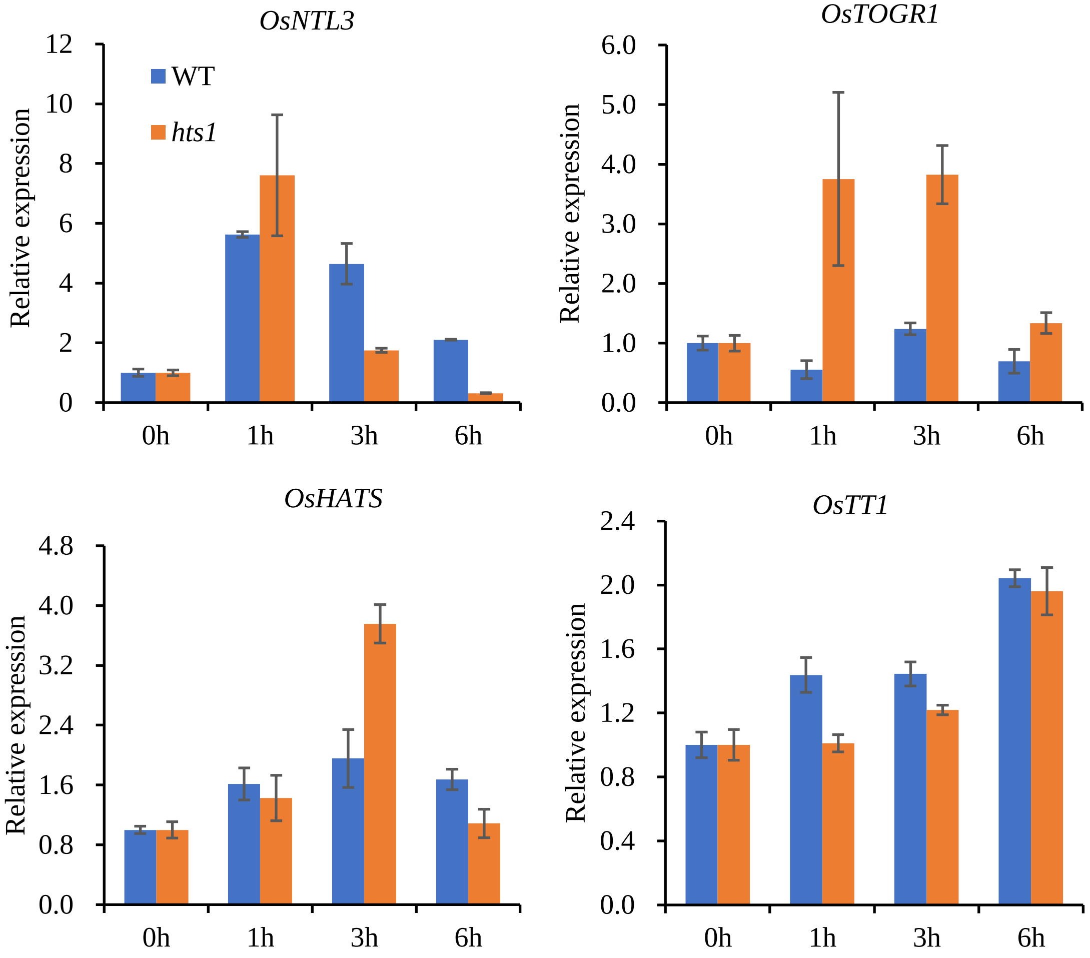

Fig. S12. Expression of rice heat-resistance-associated genes in *hts1* and WT plants under heat treatment. qRT-PCR analysis of mRNA levels of heat-resistance-associated genes, including *OsNTL3*, *OsTOGR1*, *OsHATS* and *OsTT1*, in leaves from 2-week-old wild type and mutant seedlings challenged with heat treatment (45 °C) for the indicated times. Actin (*LOC_Os03g50885*) was used as the internal standard. Data represent means ± SD (n = 3).

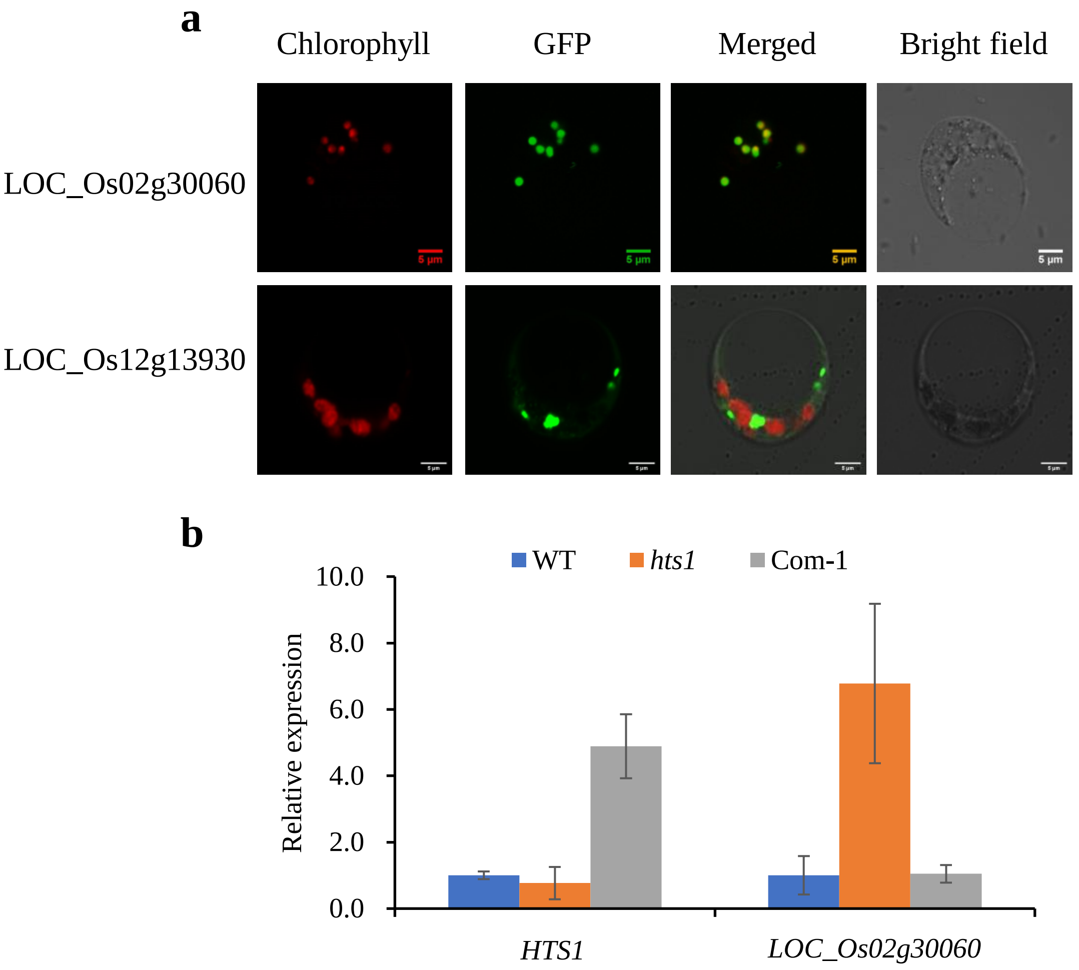

Fig. S13. Expression and subcellular localization of two rice KAR homologs. (a) Subcellular localization of LOC_Os02g30060-GFP (upper panel) and LOC_Os12g13930-GFP (lower panel) in rice protoplast cells. Left to right, GFP fluorescence, chloroplast autofluorescence, merged images and bright field images. Bar: 5 μM. (b) qRT-PCR analysis of HTS1 and *LOC_Os02g30060* expression in leaves from wild-type and mutant seedlings. Actin (LOC_Os03g50885) was used as an internal control. Data represent means ± SD (n = 3).
